# Motivational withdrawal in college English learning: a mixed-methods investigation of diminished motivational support and perceived effort ineffectiveness

**DOI:** 10.3389/fpsyg.2026.1872522

**Published:** 2026-08-03

**Authors:** Shuyun Hu, Guosheng Xu

**Affiliations:** 1 School of Humanities and Social Sciences, North University of China, Taiyuan, Shanxi, China; 2 Applied Translation Studies Center, Shandong Huayu University of Technology, Dezhou, Shandong, China

**Keywords:** diminished motivational support, exam-oriented learning setting, motivational withdrawal, perceived effort ineffectiveness, vocational English learning

## Abstract

**Introduction:**

This study investigates the latent motivational structure and qualitative manifestations of motivational withdrawal among some Chinese higher vocational college students learning College English within exam-oriented instructional contexts. Drawing on Self-Determination Theory (SDT) and Learned Helplessness Theory (LHT), the study investigates how diminished self-determined motivational support and perceived effort ineffectiveness are reflected in students’ reported experiences and observable disengagement.

**Methods:**

It employed a multiple-phase mixed-methods design involving an exploratory sample of 200 students, a confirmatory sample of an additional 200 students, and 5 English teachers from three higher vocational colleges in Jilin, Shandong, and Guangdong in China. Data from a 10-item questionnaire were analyzed through exploratory factor analysis (EFA) and confirmatory factor analysis (CFA), while qualitative data from open-ended questionnaires, a focus group interview, classroom observations, platform records, and teacher interviews were subjected to thematic analysis.

**Results:**

The findings suggested a contextually coherent two-factor pattern within the present vocational English-learning sample that was supported through both exploratory and confirmatory factor analyses conducted on separate samples. Qualitative evidence showed that these dimensions were manifested in low task meaning, weak ownership of learning, effort-nullifying beliefs, anticipated failure, and recurrent non-engagement in exam-review activities.

**Discussion:**

Rather than representing simple unwillingness, students’ disengagement was therefore interpreted as a patterned form of motivational withdrawal characterized by weakened motivational support and limited confidence in the effectiveness of effort within exam-oriented vocational English-learning contexts.

## 1 Introduction

College English curriculum within China’s higher vocational education system are expected to balance instrumental and humanistic goals (Cao et al., 2024; Wang, 2023; Yu and Liu, 2018). Within this broader mandate, however, teaching and learning in many vocational College English settings continue to be organized around examination requirements and associated review practices. Before examinations, teachers commonly highlight frequently tested content, key language points, and review priorities, making pre-exam preparation a visible part of classroom practice. Within this context, some students display recurrent disengagement during pre-exam review even when examination content and preparation requirements are clearly communicated. In this study, such disengagement refers to an observable absence of preparation behaviors, including not opening textbooks, not taking notes, and not accessing review materials during pre-exam sessions.

This problem is very obvious among vocational college students, who have often experienced long-term academic marginalization within China’s stratified education system (Gu and Schweisfurth, 2006). In English learning, the mismatch between students’ weak linguistic foundations and the difficulty of curriculum materials intensifies this vulnerability, and undermines their confidence and learning efficacy (Bandura, 1982). Under such conditions, recurrent non-engagement may reflect more than temporary unwillingness or weak discipline. It may instead indicate a patterned form of motivational withdrawal characterized by diminished motivational support and limited confidence in the effectiveness of effort. Accordingly, exam-oriented instruction is treated in this study as the contextual setting in which motivational withdrawal is examined, rather than as an independently measured cause of that withdrawal.

To investigate the internal structure of this withdrawal, the study integrates two theoretical perspectives: Self-Determination Theory (SDT) and Learned Helplessness Theory (LHT). Drawing on these perspectives, it focuses on two theoretically informed dimensions: diminished self-determined motivational support and perceived effort ineffectiveness. SDT highlights autonomy, competence, and meaningful engagement as important conditions for sustaining motivation, whereas LHT helps interpret how repeated difficulty and low perceived control may give rise to beliefs that further effort will not improve learning outcomes. Together, these perspectives provide a framework for examining how motivational withdrawal is organized and how its dimensions are manifested in students’ reported experiences and observable engagement patterns.

Accordingly, this study poses two questions: (1) What latent motivational structure characterizes motivational withdrawal among Chinese vocational English learners? (2) How are the identified motivational dimensions manifested in students’ reported experiences and observable engagement patterns? By addressing these two questions, the study seeks to provide a more empirically grounded and context-sensitive account of the internal structure of motivational withdrawal and its qualitative manifestations in vocational English learning.

## 2 Literature review

### 2.1 Exam-oriented vocational English learning and disengagement

In exam-oriented educational systems, teaching organization and students’ learning experiences are very likely to be shaped by assessment policies and review practices (Fischer et al., 2024; Ramezaney, 2014). Within Chinese higher vocational English education, exam preparation, review sessions, and predicted test content often become central classroom priorities (Hou and Huang, 2025). These practices are intended to provide clarity and direction, yet they may also reduce learning to externally imposed requirements. Previous empirical research has shown that exam-oriented instructional environments may contribute to reduced learner engagement, passive classroom participation, and instrumental learning attitudes, particularly among students with relatively weak academic foundations (Zhang, 2025). Research in vocational education has further suggested that students often experience English learning as disconnected from their future occupational goals and personal interests, thereby weakening sustained motivational involvement (Skarpaas, 2024).

On this basis, students’ disengagement may reflect a deeper weakening of motivational support and a growing sense that effort is ineffective (Jang et al., 2016; Liu et al., 2024; Maier and Seligman, 1976). Importantly, previous research has tended to examine disengagement either as a behavioral problem or as a general motivational decline, while relatively limited attention has been paid to the latent structure of motivational withdrawal and to how its constituent dimensions are manifested among vocational English learners situated within exam-oriented instructional contexts. This issue may be especially important in vocational English classrooms, where examination practices remain highly influential and students often enter with weak academic confidence.

### 2.2 Self-determination theory and motivational support

From the perspective of SDT, the motivation quality depends on the extent to which learners’ basic psychological needs for autonomy, competence, and relatedness are satisfied (Deci et al., 2017; Fakhrutdinova et al., 2024; Zhu et al., 2024; Yang et al., 2025). Recent SDT-based research has shown that need-supportive and autonomy-supportive educational environments are positively associated with students’ engagement, autonomous motivation, persistence-related learning outcomes, and psychological well-being (Yang et al., 2022; Wang et al., 2021). Conversely, instructional environments characterized by controlling teacher behavior and externally imposed learning demands may frustrate students’ autonomy, reduce autonomous motivation, and weaken their active engagement in learning (Johansen et al., 2023).

In exam-driven classrooms, however, students tend to have limited control over learning tasks, little room for meaningful choice, and repeated exposure to activities that are defined mainly by assessment demands (Patall et al., 2010; Reeve and Cheon, 2021; Zeng and Huang, 2021). Existing SDT-related research in language-learning contexts has mainly focused on general university students, learner autonomy, and motivational enhancement strategies. Comparatively less attention has been paid to how vocational college students experience diminished motivational support within exam-oriented English-learning environments characterized by repeated assessment pressure and long-term academic frustration.

### 2.3 Learned helplessness and perceived effort ineffectiveness

Diminished self-determined motivational support and perceived effort ineffectiveness may be theoretically related, although the present study does not test their temporal ordering. According to LHT, repeated failure may lead learners to believe that outcomes are beyond their control, thereby reducing their motivation and encouraging withdrawal (Liu et al., 2024; Maier and Seligman, 1976; Yue, 2025). The term “withdrawal” in this paper is used to describe a patterned combination of weakened motivational support, perceived effort ineffectiveness, and recurrent disengagement observed in the current research context. Previous empirical research has suggested that learned helplessness in educational settings is associated with students’ perceived lack of control, reduced motivation to make further effort, disengagement from learning, and lower academic performance; in language-learning contexts, students who repeatedly perceive effort as ineffective may become increasingly likely to avoid participation, and anticipate failure in advance (Ghasemi, 2021).

In the context of vocational education, many students reported repeated experiences of difficulty and unsuccessful English learning, which may contribute to relatively persistent perceptions that meaningful improvement is difficult to achieve despite effort (Han and Hamzah, 2024; Wei and Tu, 2023; Xu et al., 2021). Once this belief becomes internalized, students may stop investing effort even when external incentives remain present and even when teachers attempt to simplify review demands or provide explicit exam guidance (Darnon et al., 2024; Wang et al., 2025).

Although learned helplessness has been widely discussed in educational psychology, existing studies have often conceptualized helplessness primarily as an individual psychological outcome. Relatively limited research has examined perceived effort ineffectiveness as one dimension of a broader motivational withdrawal structure or explored how it is manifested in vocational English learners’ reported experiences and observable engagement patterns.

### 2.4 Integrating SDT and LHT in vocational English contexts

The relationship between the two dimensions can be theorized developmentally, although the present study examines their association rather than establishing temporal ordering. When classroom environments repeatedly frustrate students’ needs for autonomy and competence, motivation may lose its self-sustaining quality (Behzadnia et al., 2024; Johansen et al., 2025; Ryan and Deci, 2000). Alongside this diminished motivational support, experiences of low control, repeated setbacks, and limited success may be associated with helplessness-related beliefs that effort is ineffective (Liu et al., 2024). In other words, SDT helps explain why students may not experience English learning as something with which they can meaningfully engage, while LHT helps interpret why they may perceive further effort as unlikely to improve their outcomes. Although these perspectives permit a possible developmental interpretation, the present study does not test a temporal transition from diminished motivational support to helplessness; instead, it examines them as related dimensions of motivational withdrawal.

Together, these two dimensions provide a coherent account of motivational withdrawal in vocational English classrooms: one reflects the erosion of motivational conditions, and the other reveals the internalization of beliefs that effort is ineffective. Recent research suggests that motivational disengagement should not be understood as an isolated individual problem, but as a process shaped by the interaction between educational environments, institutional conditions, and students’ failure-related self-perceptions (Darnon et al., 2024). However, empirical attempts to integrate SDT and LHT within vocational English-learning contexts remain relatively limited. Existing research has rarely examined whether diminished self-determined motivational support and perceived effort ineffectiveness constitute distinguishable but related dimensions of motivational withdrawal or how these dimensions are manifested qualitatively in students’ learning experiences and engagement patterns.

This integrated perspective is important in exam-oriented vocational settings, where observable non-engagement may easily be interpreted as laziness, weak discipline, or a temporary lack of interest. An integrated SDT–LHT framework instead directs attention to whether students experience limited ownership of learning and doubt the effectiveness of further effort. The exam-oriented setting therefore provides an important contextual background for examining motivational withdrawal, rather than an independently measured construct whose causal effects are tested in the present study. Understanding motivational withdrawal consequently requires attention not only to observable disengagement, but also to the relationship between diminished self-determined motivational support and perceived effort ineffectiveness that may underlie it.

### 2.5 Research gap and present study

Although previous studies have examined motivational decline, disengagement, and helplessness in educational contexts, several important limitations remain. First, existing SDT-related research has primarily focused on general university students or broad language-learning populations, while vocational college students within highly exam-oriented English-learning environments remain comparatively underexamined. Second, studies grounded in learned helplessness theory have mainly emphasized psychological outcomes at the individual level, with less attention to how perceived effort ineffectiveness is situated within students’ broader motivational experiences. Third, relatively limited research has integrated SDT and LHT to investigate the latent structure of motivational withdrawal and connect quantitatively identified dimensions with their qualitative manifestations in vocational English learning.

Accordingly, the present study seeks to address these gaps by examining the motivational structure and qualitative manifestations of withdrawal among Chinese vocational English learners through an integrated SDT–LHT framework. Exam-oriented instruction is treated as the contextual background within which students’ motivational experiences and engagement patterns are examined. Specifically, the study investigates whether diminished self-determined motivational support and perceived effort ineffectiveness constitute distinguishable but related dimensions of motivational withdrawal and how these dimensions are manifested in students’ reported experiences and observable engagement patterns.

## 3 Research methods

### 3.1 Research design and participants

Methodologically, the study employed a multiple-phase mixed-methods design, in which exploratory quantitative analysis was followed by confirmatory quantitative validation and qualitative interpretation to develop a context-sensitive explanation of motivational withdrawal. Initially, 211 questionnaires were collected from students across three higher vocational colleges located in Jilin, Shandong, and Guangdong. After data screening, 200 valid questionnaires were retained for the exploratory phase of the study. Eleven questionnaires were excluded because of invalid response patterns, including identical responses across all items and excessively short completion times. The final exploratory sample involved 200 students including 75 students from Jilin, 62 from Shandong, and 63 from Guangdong. The participants included both male and female first-year vocational college students aged 18–21 from multiple disciplinary backgrounds, including engineering, business and media majors. Although participants displayed varying levels of English proficiency, most had experienced prolonged exam-oriented English instruction during secondary education and generally reported relatively weak English-learning foundations prior to entering vocational college. The exploratory sample size was considered sufficient for qualitative saturation and preliminary factor exploration. Importantly, the sample included students with varying levels of classroom participation, review engagement, and motivational orientation, rather than a predefined low-motivation subgroup. Moreover, 5 English teachers (T1-T5 (T means teacher), *n* = 2 in Jilin; *n* = 2 in Shandong; *n* = 1 in Guangdong), ensuring diversity and representativeness.

Because classroom observations were conducted only in the Shandong site, cross-site thematic stabilization was primarily assessed through questionnaire responses and student interviews from all three institutions, while observation data were used to provide contextual behavioral validation in Shandong. All participants were informed of the research purpose, procedures, and their rights, and written informed consent was obtained. Participation was voluntary. Purposive sampling was used to recruit student participants with sustained exposure to exam-oriented College English instruction. All student participants were first-year vocational college students enrolled in compulsory College English curriculum and had continuous classroom participation during the semester. Importantly, participants were not selected on the basis of low academic achievement, low English proficiency, low motivation, or prior disengagement. Rather, the exploratory sample was intended to reflect ordinary vocational college students who shared sustained exposure to exam-oriented English instruction within their institutional contexts. All teacher interviewees were interviewed through Tencent Meeting. In addition to the exploratory sample, a separate validation sample consisting of 208 newly collected questionnaires (involving engineering, business and media majors) from the same three vocational colleges was obtained for CFA analysis. After data screening, 200 valid questionnaires (69 from Jilin, 76 from Shandong and 55 from Guangdong) were retained, while 8 questionnaires were excluded because of invalid response patterns, including identical responses across all items, extremely short completion times.

### 3.2 Questionnaire development and quantitative analysis

All 200 students completed an open-ended questionnaire, and a 10-item five-point Likert scale developed from SDT (5 items) and LHT (5 items). All items were rated on a five-point Likert scale ranging from 1 (“strongly disagree”) to 5 (“strongly agree”). The questionnaire items were developed through a multi-step adaptation process. First, an initial item pool was generated based on the key constructs of SDT and LHT. Second, the items were reviewed by two senior College English teachers and one educational psychology researcher, thus conceptual clarity and contextual relevance for vocational English learners were guaranteed. The review process focused on item clarity, theoretical relevance, contextual appropriateness, and content representativeness in relation to vocational English-learning contexts. Third, a small pilot review with several students from a similar vocational context was conducted to identify ambiguous wording and improve readability. The questionnaire was originally drafted in Chinese and then translated into English for reporting purposes. To enhance translation accuracy, the English version was independently back-translated into Chinese by a bilingual researcher, and discrepancies between the original and back-translated versions were discussed and resolved through iterative revision. The resulting 10-item scale was adapted to the vocational English context and used to map students’ motivational tendencies and to test theoretical constructs statistically.

The full questionnaire items, including the original Chinese version, English translation, response anchors, and reverse-coded items, are presented in Appendix A. The scale was not intended as a fully standardized psychometric instrument, but rather as a context-specific measurement tool designed to preliminarily capture vocational college students’ motivational tendencies under exam-oriented English instruction. Accordingly, the present instrument should be understood primarily as a context-sensitive exploratory measurement tool developed to support the current mixed-methods investigation rather than as a fully validated standalone psychometric scale.

The hypothesized two-factor model consisted of five SDT-related items and five reverse-coded LHT-related items. The reverse-coded LHT indicators were modeled so that higher scores reflected lower levels of learned helplessness and more adaptive motivational responses. Thus, the LHT dimension in the present study should not be interpreted as measuring clinical helplessness directly, but rather as reflecting students’ perceived ineffectiveness of effort within exam-oriented English-learning contexts. The resulting 10-item scale was adapted to the vocational English context and used to map students’ motivational tendencies and to test theoretical constructs statistically. To enhance the psychometric rigor of the scale, this study employed both EFA and CFA. EFA was first conducted using the exploratory sample of 200 students to identify the latent structure of the questionnaire. CFA was subsequently performed using an independent validation sample consisting of 200 newly collected valid questionnaires to provide additional psychometric support for the factor structure identified in the exploratory stage.

### 3.3 Qualitative data collection

To complement the quantitative findings and examine how the two identified motivational dimensions were manifested in students’ reported experiences and observable engagement patterns, multiple qualitative data sources were collected, including open-ended questionnaires, a focus group interview, classroom observations, platform observations, and teacher interviews. The qualitative phase was conducted primarily with participants from the exploratory stage of the study, namely the initial 200-student sample used for EFA. Qualitative material concerning examination preparation and review guidance was used to situate participants’ motivational experiences and engagement patterns within the instructional context; it was not intended to operationalise exam-oriented instruction as an independent construct or to test its causal effects.

All 200 participants from the exploratory sample completed open-ended questionnaire responses alongside the Likert-scale instrument. Following qualitative data screening, 194 responses (SQ1–SQ194; SQ means student questionnaire) were retained for analysis, while 6 responses were excluded because they were blank, excessively brief, or lacked interpretable content relevant to the study. The open-ended section invited students to describe their attitudes toward English learning, exam preparation, review behavior, and perceptions of effort effectiveness within exam-oriented English classrooms. In terms of items in the open-ended questionnaire, please see Appendix B. Although the questionnaire was organized around the overall research objectives, all questions were intentionally phrased in an open-ended manner to encourage participants to describe their own learning experiences rather than confirm predefined theoretical assumptions.

In addition, one online focus group interview involving 10 students (SI1–SI10, SI means student interview) was conducted via Tencent Meeting. Participants were selected from the exploratory sample through purposive sampling to ensure that interviewees came from the same three-institution research context and had completed the questionnaire. The selection aimed to include students with ordinary exposure to exam-oriented College English instruction and to explore how different students understood exam preparation, effort, and English learning. The interview lasted approximately 90 min and explored students’ cognitive evaluations and emotional responses to behaviors such as refusing to review and ignoring high-frequency exam content, focusing on task value perceptions, the impact of past failure experiences, and interpretations of exam-oriented instruction. The interview was audio-recorded with participant consent and transcribed verbatim before analysis. The focus group followed a semi-structured discussion format guided by open-ended prompts concerning exam preparation, perceived effort ineffectiveness, classroom engagement, and motivational experiences. Before formal data collection, the focus group interview guide was reviewed by two experienced College English teachers and one educational psychology researcher to improve question clarity, contextual appropriateness, and neutrality. Major revisions were made to reduce potentially leading expressions and make the sentence meaning clearer. Although the interview guide was informed by the overall research framework, the questions were intentionally phrased to encourage participants to describe their own learning experiences, classroom practices, and perceptions in their own words rather than asking them to confirm predefined theoretical relationships. During interviews, participants were encouraged to introduce issues they considered important, and follow-up questions were used primarily for clarification rather than confirmation of theoretical expectations. Probing questions were used throughout the interview to encourage clarification and interaction among participants. In terms of the focus group interview guide, please see Appendix C. The focus group interview generated approximately 16,000 Chinese characters in a clean-read transcript (approximately 15 Microsoft Word pages, SimSun, 10.5-point font, single-line spacing). No separate follow-up clarification interviews were conducted because issues requiring clarification were addressed during the interview through iterative probing, summarizing and confirming participants’ responses, and participant-to-participant discussion.

Classroom observations (CO1–CO10, CO means classroom observation) were carried out across ten class sessions involving 26 students (from the exploratory sample) from one intact class in Shandong. Observation focused on attendance, attention, note-taking, non-academic behavior, participation, and engagement during ordinary instruction and pre-exam review sessions. Classroom observation also included SuperStar platform observation (CO9 and CO10), which documented assignment submission, online activity, and interaction frequency during the 2 weeks before the exam. CO1–CO8 consisted primarily of on-site classroom observations, whereas CO9 and CO10 combined on-site observation with platform-record analysis. Observational records were archived chronologically to facilitate later cross-analysis with interview and questionnaire data.

To incorporate teachers’ perspectives, in-depth semi-structured interviews were conducted via Tencent Meeting with five English teachers from three vocational colleges (TI1–TI5, TI means teacher interviewee), all of whom had more than 5 years of teaching experience. Each interview lasted approximately 20–60 min. Before formal data collection, the teacher interview guide was reviewed by the same two experienced College English teachers and one educational psychology researcher. Major revisions were made to improve question clarity, contextual appropriateness, and neutrality, while reducing potentially leading expressions. Although the guide was informed by the overall SDT–LHT framework, the interview questions were designed to invite teachers to describe their classroom observations and professional experiences rather than confirm predefined theoretical assumptions. Interviews explored students’ exam-preparation behaviors, teachers’ observations of disengagement during review activities, perceived effort ineffectiveness, and the ways in which motivational withdrawal became visible within exam-oriented instructional contexts. In terms of the teacher interview (see Appendix D), questions were reviewed and refined before formal data collection to improve clarity and avoid wording that might encourage teachers to interpret students’ experiences solely through predefined motivational constructs. Teachers were instead invited to describe their own classroom observations and professional interpretations before any theoretically informed probing was introduced. The teacher interviews generated approximately 29,000 Chinese characters in a clean-read transcript (approximately 28 Microsoft Word pages, SimSun, 10.5-point font, single-line spacing). Teachers frequently described students remaining disengaged during exam-oriented review even when preparation guidance was explicit; these accounts were used to contextualize the qualitative manifestations of motivational withdrawal.

### 3.4 Qualitative data analysis and trustworthiness

All qualitative data, including interview transcripts, open-ended questionnaire responses, classroom observation records, platform observation records, and teacher interviews, were imported into NVivo 14 for organization and analysis. Interview recordings were transcribed verbatim before coding. To enhance familiarity with the data and improve analytical consistency, the researcher repeatedly reviewed transcripts alongside classroom observation records, platform data, and reflexive memos throughout the analysis process.

The study adopted a theory-informed thematic analysis guided primarily by SDT and LHT. Although SDT and LHT informed the overall analytical framework, they were not presented to participants during data collection and did not determine the wording or sequence of interview questions. Theoretical concepts were used primarily during analysis as sensitizing concepts rather than as interview prompts. When multiple plausible interpretations were possible, alternative explanations were explicitly compared before assigning a final analytic category. Interpretations were evaluated against the immediate conversational context, surrounding extracts, and evidence from questionnaires, classroom observations, platform records, and teacher interviews. A theoretically informed interpretation was retained as the primary thematic interpretation only when it was supported across multiple qualitative sources and provided the most coherent explanation of the broader motivational pattern observed in the dataset. Initial coding combined deductive attention to motivational support and helplessness-related patterns with inductive attention to recurring contextual meanings emerging from the data. Codes were iteratively refined through repeated comparison across focus group interview, questionnaires, classroom observations, platform records, and teacher interviews. Related codes were subsequently grouped into broader thematic categories through iterative theme development. Themes were continuously reviewed and refined to ensure conceptual coherence, explanatory relevance, and consistency across multiple qualitative data sources. An initial coding framework was first developed based on sensitizing concepts derived from SDT and LHT, including perceived autonomy, competence frustration, effort ineffectiveness, and withdrawal-related behaviors. During repeated coding cycles, additional inductive codes emerging from participants’ descriptions were incorporated into the evolving codebook. Similar codes were merged, refined, or reorganized through iterative comparison across interviews, questionnaires, classroom observations, and teacher reports. The final thematic structure was generated through repeated movement between coded extracts, broader thematic categories, and the overall dataset. In terms of the coding process, code refinement and theme development, please see Appendix E.

Before the thematic structure was finalized, the provisional themes, thematic boundaries, and supporting extracts were discussed with an experienced qualitative research colleague (with the title of Professor) familiar with vocational English education. These discussions focused on whether the proposed themes adequately represented the data, whether alternative thematic organizations remained plausible, and whether theme names and boundaries accurately reflected the coded material. Final decisions regarding theme refinement remained the responsibility of the researcher following these discussions. The purpose of these discussions was to challenge emerging interpretations, consider alternative explanations, and strengthen the credibility and analytical transparency of the final thematic structure.

Attention was paid to patterns that did not fully align with predefined theoretical expectations. Consequently, theoretical categories were treated as interpretive explanations rather than predetermined labels, and coding decisions remained open to contextual revision throughout the analytic process. For example, some students framed English learning as pragmatically irrelevant to their future work rather than explicitly expressing helplessness-related beliefs. Such responses were retained during coding and interpreted cautiously to avoid excessive theoretical reductionism. This approach helped maintain sensitivity to contextual variation and reduced the risk of over-interpreting all disengagement solely through the SDT–LHT framework. Accordingly, not all expressions of disengagement were automatically interpreted as direct indicators of motivational withdrawal, and alternative contextual explanations were considered throughout the analysis process.

To enhance analytical trustworthiness, triangulation across multiple qualitative and quantitative data sources was employed throughout the analysis process. Emerging interpretations and thematic boundaries were repeatedly cross-checked against all qualitative and quantitative data sources to reduce selective interpretation and strengthen analytical consistency. Reflexive memos were also used to document analytic decisions, emerging interpretations, potential researcher assumptions, and coding reflections during theme development. Following this collaborative analytic review, reflexive memos were used to document the rationale for retaining, revising, or merging themes throughout the refinement process.

Formal intercoder reliability procedures were not employed because the study followed an interpretivist and reflexive thematic analysis orientation. Within this interpretive framework, coding was understood as a reflexive and theoretically informed analytic process. Coding consistency was strengthened through repeated data immersion, iterative code refinement, and continuous comparison across multiple data sources. In addition, portions of the coded dataset were revisited at multiple stages of analysis to examine coding stability and thematic consistency across time and data sources. Qualitative saturation was considered achieved when no substantially new motivational patterns, explanatory themes, or behavioral variations emerged across successive rounds of coding and cross-data comparison. Saturation was assessed iteratively across interviews, questionnaires, classroom observations, and teacher reports. The qualitative analysis was conducted based on 194 valid open-ended questionnaire responses together with interview, observation, and teacher-interview data. Formal member-checking procedures were not implemented because the study adopted an interpretivist and reflexive thematic analysis orientation, within which qualitative interpretation was understood as a theoretically informed analytical process rather than a direct reproduction of participants’ self-understandings. Nevertheless, clarification, follow-up probing, and interpretive cross-checking were continuously employed during interviews and analysis to reduce misunderstanding and preserve contextual meaning. In addition, emerging interpretations were repeatedly compared against participants’ original statements, classroom observations, platform records, and teacher reports to strengthen interpretive credibility.

Although the qualitative phase was conducted primarily with participants from the exploratory sample used in the EFA stage, the subsequent CFA was conducted using an independent validation sample consisting of 200 newly collected valid questionnaires.

Because the present study generally employed a cross-sectional rather than longitudinal design, the findings should not be interpreted as demonstrating recurring developmental trajectories of helplessness over time. Instead, the study primarily captures participants’ motivational tendencies, disengagement patterns, and self-reported perceptions within a particular institutional and examination context.

### 3.5 Researcher positionality and data integration

The researcher simultaneously served as classroom instructor and observer during the classroom observation process. While this dual role enabled sustained access to naturally occurring classroom interactions and long-term engagement with participants, it may also have influenced students’ behaviors under existing power relations and grading expectations. To reduce this influence, students were informed in advance that participation and observational findings would not affect academic grades or classroom evaluation. Observational attention was directed primarily toward recurring behavioral patterns. Nevertheless, the possibility that students modified certain classroom behaviors because they were aware of the researcher’s dual instructional and observational role cannot be fully excluded.

Reflexive memos were used throughout the study to critically examine the researcher’s positionality, assumptions, emotional responses, and potential interpretive bias during both data collection and analysis. The researcher also repeatedly cross-checked observational interpretations against questionnaire responses, student interviews, teacher interviews, and platform records to reduce the risk of relying excessively on single-source interpretation.

Consistent with Creswell and Plano Clark (2023), the integration of qualitative and quantitative data enhanced theoretical coherence through cross-validation and supported the construction of a multidimensional explanatory framework for ineffective motivation in vocational English learning.

Integration occurred across multiple stages of the study. At the design stage, the study combined exploratory quantitative analysis, confirmatory quantitative validation using an independent sample, and qualitative data collection to examine motivational withdrawal from multiple methodological perspectives. At the analysis stage, qualitative data were analyzed through theory-informed thematic analysis with reflexive procedures, while quantitative analysis was used to identify and validate broader motivational dimensions. Integration occurred primarily at the interpretation stage, where qualitative themes were interpreted alongside quantitative patterns to develop a context-sensitive explanation of motivational withdrawal in vocational English-learning contexts.

**Figure 1:**
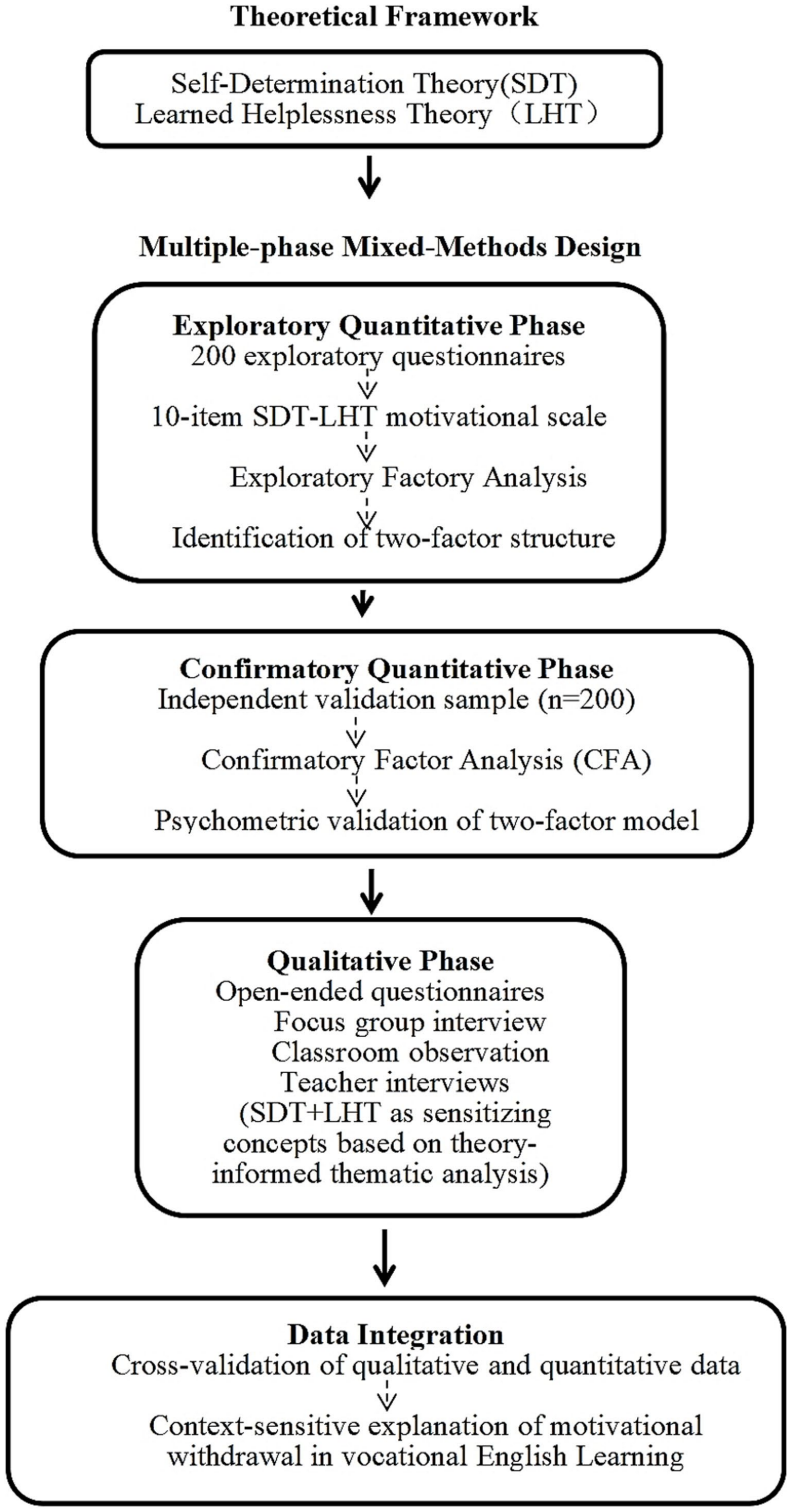
Research methodological framework.

The methodological framework is as followed (Figure 1):

## 4 Results

### 4.1 Factor-analytic examination of the two-dimensional structure of motivational withdrawal

To examine the latent structure of motivational withdrawal among vocational English learners situated within exam-oriented instructional contexts, the 10-item questionnaire was subjected to a two-stage factor-analytic procedure. EFA was first conducted using the exploratory sample to identify the underlying dimensional structure of the scale, followed by CFA using an independent validation sample to test the stability and adequacy of the extracted model. This two-stage procedure was employed to separate exploratory factor identification from subsequent psychometric examination of the proposed structure. Together, these analyses examined whether responses to the questionnaire could be represented by a coherent two-factor structure and how the dimensions associated with motivational withdrawal were organized.

#### 4.1.1 EFA results

The EFA was conducted on the 10-item revised questionnaire using data from 200 vocational college students via SPSS 23. Prior to extraction, the suitability of the data for factor analysis was confirmed. As Table 1 shows The Kaiser–Meyer–Olkin (KMO) measure of sampling adequacy was 0.902, and Bartlett’s test of sphericity was significant, *χ*^2^(45) = 939.534, *p* < 0.001, showing that the correlation matrix was appropriate for factor analysis. Principal axis factoring (PAF) was employed with Promax rotation, given the assumption that the latent dimensions of motivational withdrawal were conceptually related. Listwise deletion was used for missing-data handling before factor extraction. The extraction criterion was based on eigenvalues greater than 1.00, and factor loadings below 0.40 were suppressed in the rotated pattern matrix.

**Table 1:** Exploratory factor analysis results for the 10-item motivational withdrawal scale (*N* = 200).

Item	Factor 1: perceived effort ineffectiveness (LHT)	Factor 2: self-determined motivational support (SDT)	Communality (h^2^)
LHT5R	0.733		0.563
LHT4R	0.723		0.512
LHT3R	0.716		0.551
LHT2R	0.692		0.520
LHT1R	0.685		0.449
SDT5		0.917	0.658
SDT2		0.700	0.537
SDT3		0.670	0.508
SDT4		0.664	0.564
SDT1		0.564	0.593
Initial eigenvalue	5.176	1.178	
Variance explained (%)	47.253	7.320	
Cumulative variance (%)	47.253	54.573	

KMO = 0.902. Bartlett’s test of sphericity: χ^2^(45) = 939.534, p < 0.001. Extraction method: Principal axis factoring (PAF). Rotation method: Promax with Kaiser normalization. Factor loadings below 0.40 were suppressed. The correlation between the two retained factors was r = 0.694. LHT items were reverse-coded prior to factor analysis so that higher scores reflected lower levels of learned helplessness and more adaptive motivational responses.

A clear two-factor solution was yielded. As Table 1 reveals, the first two factors had eigenvalues above 1.00 and together accounted for 54.573% of the total extracted variance. Specifically, the initial eigenvalues of Factor 1 and Factor 2 were 5.176 and 1.178, respectively. After extraction, the two retained factors accounted for 47.253 and 7.320% of the variance, respectively. More specifically, Factor 1 represented effort-related beliefs derived from LHT, with lower scores indicating greater perceived effort ineffectiveness, whereas Factor 2 represented self-determined motivational support, with lower scores indicating diminished support. In the pattern matrix, the five learned helplessness items loaded strongly on Factor 1, with loadings ranging from 0.685 to 0.733. The five self-determination items loaded on Factor 2, with loadings ranging from 0.564 to 0.917. The extracted communalities ranged from 0.449 to 0.658, indicating that all items shared an acceptable amount of variance with the retained factor solution. No substantial cross-loadings above the reporting threshold were observed in the rotated pattern matrix, suggesting a relatively clean factor structure.

Additionally, the factor correlation matrix showed that the two factors were moderately to strongly correlated (*r* = 0.694), showing that they were closely related but still empirically distinguishable. Overall, the EFA suggested that motivational withdrawal in this sample was not organized around four empirically separable dimensions, but around two closely related core domains: diminished self-determined motivational support and perceived effort ineffectiveness.

#### 4.1.2 CFA results

To further examine the adequacy of the two-factor structure identified in the exploratory stage, CFA was conducted using an independent validation sample consisting of 200 newly collected valid questionnaires. This independent validation procedure aimed to provide additional psychometric support for the proposed motivational structure.

CFA was performed using AMOS 23 with maximum likelihood estimation. Although the questionnaire employed five-point Likert-scale items, the variables demonstrated acceptable univariate normality, with skewness and kurtosis values remaining within acceptable ranges. Therefore, the Likert-scale items were treated as approximately continuous indicators for model estimation. The hypothesized two-factor model consisted of five SDT-related items representing diminished self-determined motivational support and five reverse-coded LHT-related items. The reverse-coded LHT indicators were modeled so that higher scores reflected lower levels of learned helplessness and more adaptive motivational responses.

As the Table 2 shows, the CFA results indicated acceptable model fit: *χ*^2^(34) = 35.673, *p* = 0.390, suggesting that the proposed two-factor structure demonstrated adequate consistency with the observed data. The latent correlation between SDT and LHT was 0.631, indicating a moderate association between weakened motivational support and helplessness-related beliefs while still supporting conceptual distinction between the two constructs.

**Table 2:** CFA results for the two-factor motivational withdrawal model.

Construct	Item	Unstandardized loading	Standardized loading	CR	AVE
SDT	SDT1	1.000	0.673	0.83	0.50
	SDT2	0.980	0.718		
	SDT3	1.097	0.723		
	SDT4	1.139	0.799		
	SDT5	0.905	0.614		
LHT	LHT1R	1.000	0.675	0.80	0.45
	LHT2R	1.089	0.692		
	LHT3R	0.879	0.666		
	LHT4R	1.002	0.704		
	LHT5R	0.869	0.602		

Model fit indices: *χ*^2^(34) = 35.673, p = 0.390; latent correlation between SDT and LHT = 0.631.

LHT items were reverse-coded prior to CFA so that higher scores reflected lower levels of learned helplessness and more adaptive motivational responses.

All factor loadings were statistically significant (*p* < 0.001). The unstandardized factor loadings ranged from 0.869 to 1.139, while the standardized factor loadings ranged from 0.602 to 0.799. Specifically, SDT-related items showed standardized loadings ranging from 0.614 to 0.799, whereas LHT-related items ranged from 0.602 to 0.704. These results suggested that all items contributed meaningfully to their respective latent constructs without substantial cross-loading concerns.

To further evaluate convergent reliability, composite reliability (CR) and average variance extracted (AVE) were calculated based on the standardized factor loadings. The SDT dimension demonstrated acceptable internal consistency (CR = 0.83) and adequate convergent validity (AVE = 0.50). Similarly, the LHT dimension showed acceptable composite reliability (CR = 0.80) and convergent validity (AVE = 0.45). The AVE of 0.45 is acceptable, as Fornell and Larcker (1981) argue, convergent validity may still be considered adequate when composite reliability is satisfactory despite AVE values below 0.50. Given the exploratory and context-specific nature of the scale, these results were considered acceptable preliminary psychometric evidence rather than definitive scale validation.

Overall, the CFA findings provided additional support for the two-dimensional motivational structure identified in the exploratory stage, while the scale should still be interpreted cautiously as a context-sensitive measurement tool developed specifically for vocational English-learning settings. Because the scale was developed specifically for the present vocational English-learning context and was tested using relatively limited regional samples, the findings should be interpreted as preliminary contextual psychometric evidence.

### 4.2 Descriptive motivational tendencies in the validation sample

Following the confirmation of the two-factor structure, descriptive analysis was conducted using the independent CFA validation sample to examine students’ overall motivational tendencies in exam-oriented English learning contexts. Overall, both dimensions demonstrated relatively low mean scores across items, suggesting generally weak motivational endorsement among participants.

For the self-determined motivational support (SDT) dimension, as Table 3 shows, the five items showed consistently low mean values, ranging from 2.29 to 2.40 on a five-point scale. Among them, SDT5 demonstrated the highest mean score (*M* = 2.40, SD = 1.20), whereas SDT4 showed the lowest (*M* = 2.29, SD = 1.16). The remaining items displayed similarly low levels. These findings suggest that many students reported limited self-determined motivational support, weak personal investment in English learning, and reduced active engagement within exam-oriented instructional settings.

**Table 3:** Descriptive statistics of the two motivational dimensions in the CFA validation sample.

Dimension	Item	Mean	SD	Min	Max	Skewness	Kurtosis
SDT	SDT1	2.36	1.21	1	5	0.432	−0.935
	SDT2	2.34	1.11	1	5	0.388	−0.783
	SDT3	2.35	1.23	1	5	0.515	−0.770
	SDT4	2.29	1.16	1	5	0.511	−0.661
	SDT5	2.40	1.20	1	5	0.560	−0.584
LHT	LHT1R	2.26	1.17	1	5	0.421	−0.988
	LHT2R	2.29	1.25	1	5	0.614	−0.718
	LHT3R	2.22	1.05	1	5	0.543	−0.447
	LHT4R	2.34	1.13	1	5	0.298	−0.905
	LHT5R	2.34	1.14	1	5	0.514	−0.623

LHT items were reverse-coded. Higher scores reflected lower levels of learned helplessness and more adaptive motivational responses.

A similar tendency was observed in the learned helplessness (LHT) dimension. Although the LHT items were reverse-coded, the mean values remained relatively low, ranging from 2.22 to 2.34. LHT3R demonstrated the lowest mean score (*M* = 2.22, SD = 1.05), while LHT4R showed the highest (*M* = 2.34, SD = 1.13). The relatively low reverse-coded scores suggest that many students exhibited helplessness-related tendencies and limited confidence in the effectiveness of effort within English-learning contexts.

Across both dimensions, the standard deviations were moderate, indicating individual variation, whereas the low mean scores reflected generally weak self-determined motivational support and stronger perceptions that effort was ineffective. Taken together, these descriptive findings further support the broader interpretation that motivational withdrawal among vocational English learners involves both weakened self-determined engagement and expectations of ineffective effort. At the same time, the findings should not be interpreted as suggesting that all vocational college students experienced motivational withdrawal uniformly. Although low motivational endorsement represented a prominent overall tendency in the present sample, individual variation remained visible across both dimensions, indicating that some students still retained partial engagement, instrumental motivation, or relatively adaptive motivational orientations toward English learning.

### 4.3 Qualitative manifestations of the two-factor structure

**Figure 2:**
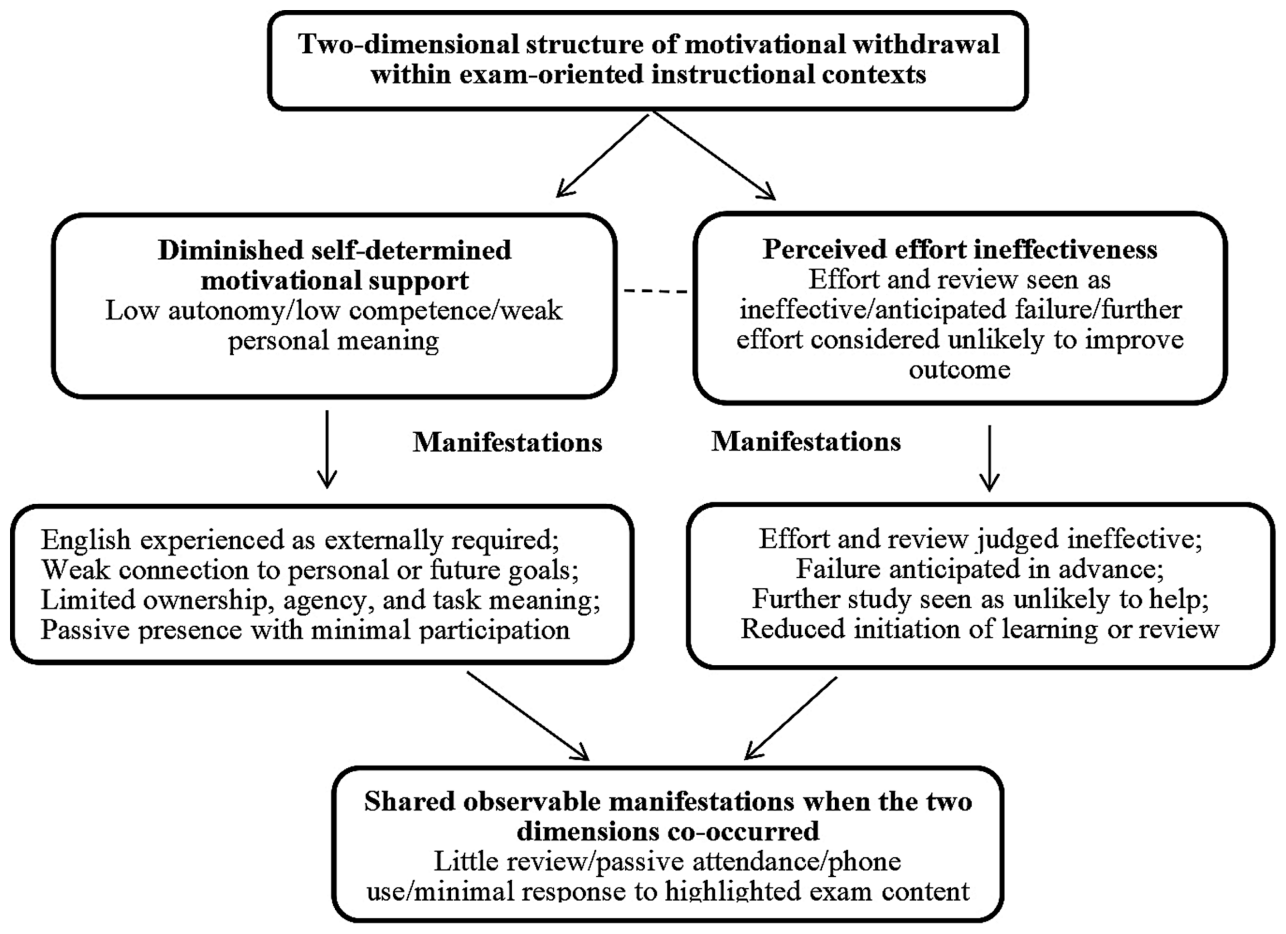
Contextual representation of the two dimensions of motivational withdrawal and their qualitative manifestations within exam-oriented instructional contexts.

Contextual representation of the two dimensions of motivational withdrawal and their qualitative manifestations within exam-oriented instructional contexts are illustrated in Figure 2.

#### 4.3.1 Diminished self-determined motivational support

Qualitative evidence from multiple data sources showed that diminished self-determined motivational support was recurrently manifested in many students’ accounts and engagement patterns, although some responses reflected pragmatic, curriculum-related, or future-oriented concerns that could not be interpreted solely as motivational withdrawal. Across open-ended questionnaires, focus group interview, classroom observations, and teacher interviews, students who demonstrated disengagement did not simply appear uninterested in English; rather, they frequently described English learning as something externally imposed, weakly connected to their own goals, and lacking personal meaning. This pattern was reflected not only in what students said about English, but also in how they participated in class and responded to exam-oriented review activities.

In the open-ended questionnaires, many students thought English was irrelevant to their present lives and future plans. Some responses described English as “meaningless,” because they did not experience it as meaningful in relation to their own trajectories. Statements such as “I’m not going abroad, so English is useless to me” (SQ127) and “No one in my family speaks English, and no one requires me to learn it” (SQ41) suggest that English learning was often detached from students’ immediate social worlds and future expectations. Similar responses also showed that review materials and exam preparation were not regarded as worthwhile learning opportunities, but as routine obligations with little personal significance (SQ8, SQ26, S185.). Importantly, such responses were not interpreted solely as evidence of motivational withdrawal. In some cases, students (SQ12, SQ18, SQ40.) appeared to evaluate English pragmatically in relation to perceived occupational relevance, future employment expectations, or immediate life priorities rather than expressing generalized helplessness toward learning itself.

Interview data reinforced this pattern. Several students described themselves as attending class without actually identifying with the learning process. One student stated, “I only attend English classes because the teacher is engaging, not because I want to learn English” (SI9). Another reported, “I want to sleep, and play games. I feel depressed and bored here, but I have to stay here” (SI7). These accounts suggest that classroom presence did not necessarily indicate motivational commitment. In this sense, attendance functioned more as compliance with institutional routine than as evidence of active learning involvement.

Classroom observations showed the same tendency in behavioral form. During ordinary lessons and pre-exam review sessions, some students remained seated in class but did not open textbooks, take notes, or respond to teachers’ guidance (CO1, CO5, CO7). In several observations, students were seen using mobile phones, chatting, or resting their heads on desks while review materials were being explained (CO2, CO4, CO5, CO6, CO7, CO8). These behaviors suggested a lack of active endorsement of classroom tasks. During sessions in which teachers narrowed the examination scope and highlighted likely test content, some students did not treat these materials as personally worthwhile.

Teacher reports provided further confirmation. Several teachers (TI1, TI3, TI4) observed that students often did not reject English openly, but instead treated it as something they had to sit through rather than something they wished to engage with. Teachers (TI1, TI2) also noted that many students perceived English as unrelated to future work or life plans, which reduced their sense of involvement even when instructional support was provided. However, some teachers (TI1, TI4) also noted that a very small of students continued to display instrumental or achievement-oriented motivation, particularly those preparing for transfer examinations, future employment requiring basic English proficiency, or certificate-related goals. These students were more likely to participate in review activities and maintained a clearer sense of purpose toward English learning.

The triangulated qualitative data suggest that diminished self-determined motivational support represented a prominent explanatory manifestation within the present sample, although some students’ disengagement also appeared to reflect pragmatic educational priorities or selective forms of participation.

#### 4.3.2 Perceived effort ineffectiveness and failure expectation

Triangulated qualitative evidence suggested that many students’ disengagement was associated with recurring perceptions of effort ineffectiveness and anticipated academic difficulty within English learning contexts. Across these data sources, students were not simply portrayed as unwilling to study or lacking discipline. Rather, they repeatedly described English learning as an area in which effort had little effect, review was unlikely to help, and failure was anticipated in advance. This pattern suggests that disengagement was often grounded in a belief that further investment would not meaningfully improve performance. Accordingly, references to exam preparation and review guidance in the analysis below are treated as contextual features within which perceived effort ineffectiveness was manifested, rather than as evidence that exam-oriented practices caused motivational withdrawal.

In the open-ended questionnaires, students frequently linked non-participation to the perceived uselessness of effort. Some responses directly expressed a sense of futility, such as “I used to memorize it, but I could never remember it, so now I just give up” (SQ17). Others framed teachers’ review support as ineffective from the outset. For example, one student wrote, “Even if the teacher gives us the questions, it’s useless; we cannot solve them even after looking at them” (SQ131). These responses may indicate that students did not simply dislike review activities, and they often regarded them as incapable of changing outcomes.

The student interviews reinforced this pattern and revealed how such beliefs operated at the level of self-perception. Interviewees frequently used phrases such as “I cannot remember,” “reading is useless,” and “I get annoyed when reading the book,” linking avoidance not to indifference alone but to anticipated ineffectiveness. One student stated, “Every time I pick up the vocabulary list, my mind goes blank, and playing phone is much easier” (SI4), while another claimed, “No matter how hard I try, I will not pass; I just do not have that IQ” (SI7). These comments suggest that some students had perceived repeated struggle as evidence of a fixed personal limitation. In such accounts, the problem was no longer a single difficult task, but an expectation that trying itself would only confirm failure.

Classroom observation provided behavioral confirmation of these narratives. During review-oriented sessions, including lessons close to the exam period (CO9, CO10), some students did not begin preparation while teachers distributed high-frequency test content and explained key points in detail. Instead, they remained passive, continued using mobile phones, rested their heads on desks, or disengaged from the task. In one observation, a student responded to the teacher’s explanation by saying, “You can keep explaining, but I will not get it” (CO9). This kind of response may indicate that the refusal to engage was not merely delay or distraction, but was tied to a prior conviction that understanding was unattainable. Classroom behavior therefore aligned closely with the self-reports found in the questionnaires and interviews.

Platform observation showed a similar tendency. Although some students maintained formal attendance and remained nominally present in the online learning system, their actual participation was limited (CO9, CO10). In the final pre-exam period, low submission rates and minimal interaction with learning materials provided observable manifestations of non-engagement within the exam-oriented instructional context (CO9, CO10).

Teacher interviews provided an additional perspective that was consistent with the student data. Several teachers (TI1, TI4, TI5) reported that students often responded to review support with comments implying that preparation was pointless. Teachers also reported that some students expressed low confidence in their ability to improve and regarded concentrating on key exam content as unlikely to change their outcomes (TI2, TI4). From the teachers’ perspective, these responses reflected more than simple laziness and were observed during sessions in which the review burden had been reduced and exam priorities made explicit.

Taken together, the triangulated data showed that failure expectations and effort-nullifying beliefs recurred across students’ accounts and observed engagement patterns. Their non-engagement therefore appeared not as simple unwillingness, but as the behavioral manifestation of perceived effort ineffectiveness. Nevertheless, the data also suggested that helplessness-related responses were not equally seen across all participants. Some students appeared to disengage selectively or pragmatically, indicating that motivational withdrawal represented a prominent but non-universal pattern within the present context.

### 4.4 Integrated pattern of dimensions and observable disengagement

These findings should be interpreted as contextually situated patterns emerging from the present institutional and participant sample. The quantitative results supported two related dimensions associated with motivational withdrawal—diminished self-determined motivational support and perceived effort ineffectiveness—while the qualitative data illustrated how these dimensions were manifested in recurring classroom and review-related behaviors. Across the integrated evidence, qualitative manifestations of the two dimensions frequently co-occurred in students’ accounts and observed participation patterns, although the findings do not establish a causal or temporal relationship between them.

Diminished self-determined motivational support was manifested in students’ weak personal identification with English learning and limited sense of ownership over learning and preparation activities. Across questionnaires, interviews, and teacher reports, many students frequently described English as unrelated to their future plans, lacking practical meaning, or worth attending only because of the teacher rather than the subject itself. Meanwhile, perceived effort ineffectiveness was manifested in repeated references to the limited value of further effort, anticipated failure, and reduced willingness to initiate learning or review activities. Students described studying as “useless,” stated that they “could not remember,” and expressed the view that teacher-provided key points would not improve their outcomes. These two strands of evidence appeared repeatedly across open-ended questionnaires, a focus group interview, teacher interviews, classroom observations, and platform observation.

When qualitative manifestations of the two dimensions co-occurred, a recurring pattern of non-engagement became visible across the data sources. Across the qualitative material, accounts of weak personal meaning and doubts about the effectiveness of effort were frequently accompanied by descriptions or observations of limited review, minimal engagement with highlighted content, physical attendance without meaningful participation, and the substitution of mobile phone use for learning activity. Classroom and platform observation further showed that formal presence did not necessarily correspond to active engagement: some students remained in the classroom or online system while withdrawing from review behavior in practice. In this integrated pattern, English learning was frequently experienced as a formal instructional requirement. Overall, the combined findings show that observable disengagement in this study corresponded to the joint presence of low self-determined motivational support and perceived effort ineffectiveness.

**Figure 3:**
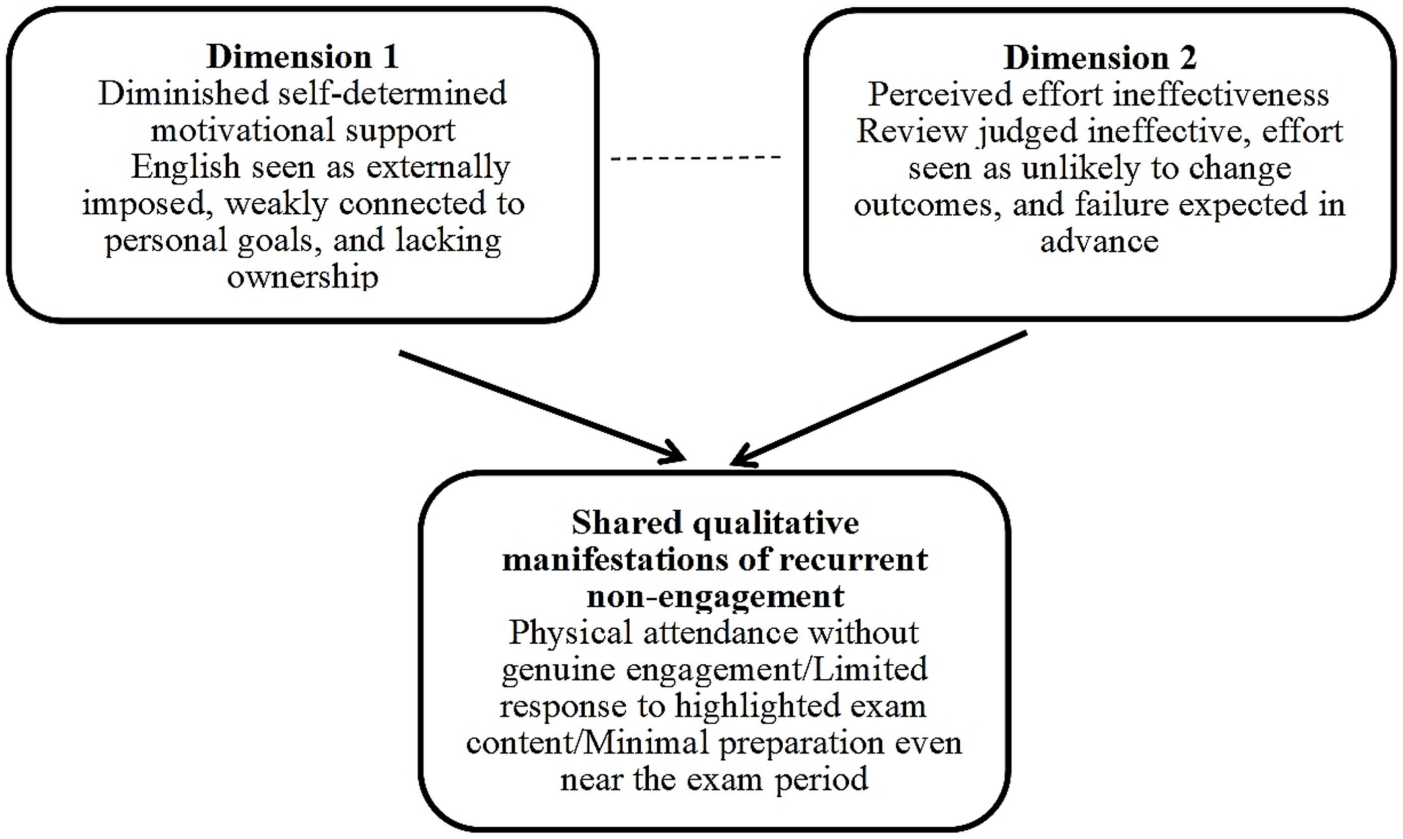
Integrated disengagement pattern across quantitative and qualitative evidence.

The integrated pattern emerging across the quantitative and qualitative evidence is summarized in Figure 3.

## 5 Discussion

### 5.1 Reframing disengagement as motivational withdrawal

The study aimed to examine whether vocational college students’ disengagement from English learning in exam-oriented classrooms could be explained as more than simple unwillingness, and the findings support such a reinterpretation. Across the quantitative and qualitative results, students’ refusal to review could not be explained by laziness, weak discipline, or temporary loss of interest. Disengagement was better understood as a form of motivational withdrawal structured by two interrelated dimensions: diminished self-determined motivational support and perceived effort ineffectiveness. The findings therefore support a reframing of disengagement in terms of its underlying motivational structure and qualitative manifestations, rather than providing a direct explanation of why exam-based incentives may lose their motivating function.

The contextual pattern identified in the study is consistent with literature suggesting that exam-oriented environments may reduce learning to externally imposed demands rather than meaningful engagement (Patall et al., 2010; Reeve and Cheon, 2021). In this sense, the findings align with SDT, which emphasizes that sustained engagement depends on the support of autonomy, competence, and relatedness (Deci et al., 2017), and with research showing that disengagement often reflects frustrated psychological needs (Jang et al., 2016). Although motivational withdrawal emerged as a prominent interpretive pattern in the present study, some students still demonstrated selective engagement, instrumental motivation, or achievement-oriented participation, particularly when English learning was connected to concrete future goals or examination requirements.

### 5.2 The significance of the two-factor structure

A key contribution of the study lies in its confirmation of a two-factor structure of motivational withdrawal. In the study, many participants’ experiences were organized around two relatively coherent motivational domains: weakened self-determined motivational support and perceived effort ineffectiveness. This is important because prior discussions of demotivation in exam-driven language education often identify multiple risk factors, such as task difficulty, low confidence, external pressure, and negative prior experience, without clearly showing how these are psychologically organized. The present results suggest that, in vocational English classrooms, these pressures converge into two core dimensions. Importantly, this two-factor structure was not only identified through EFA but was also supported through CFA using an independent validation sample, thereby providing additional evidence for the structural coherence of the proposed motivational framework. The finding clarifies the internal organization of motivational withdrawal among learners situated within an exam-oriented vocational English context. It can also be read alongside Ryan and Deci (2000), whose work suggests that participation maintained mainly by external structure differs fundamentally from participation grounded in personal endorsement. The qualitative findings further suggest that participation experienced primarily as externally required may constitute one manifestation of a broader pattern of motivational withdrawal.

### 5.3 The relationship between diminished motivational support and perceived effort ineffectiveness

The quantitative results showed that the two dimensions were closely related but empirically distinguishable, while the qualitative findings illustrated how their manifestations frequently co-occurred in students’ accounts and engagement patterns. Although the theoretical literature permits a possible developmental interpretation of this relationship, the present cross-sectional study does not establish a temporal progression from diminished motivational support to perceived effort ineffectiveness.

Students’ accounts included descriptions of English as irrelevant, externally imposed, or disconnected from their own futures. These responses reflect weakened agency, low personal endorsement, and reduced ownership of learning, all of which are central concerns within SDT (Ryan and Deci, 2000; Deci and Ryan, 2012). Alongside these descriptions, students also expressed effort-nullifying beliefs: that studying was useless, that review was unlikely to help, and that failure was already likely. These beliefs represented more than simple indifference because they reflected limited confidence that further effort could improve learning outcomes. This pattern echoes Liu et al. (2024), who show that effort–reward imbalance can foster learned helplessness and reduce learning engagement.

The present findings suggest a theoretically coherent association between the two dimensions. Diminished self-determined motivational support reflects a weakened basis for personally endorsed and self-sustaining engagement, whereas perceived effort ineffectiveness reflects doubts that further investment can change learning outcomes. Longitudinal research would be required to determine whether diminished motivational support precedes or develops into helplessness-related beliefs over time.

### 5.4 Motivational withdrawal within exam-oriented instructional contexts

Exam-oriented instruction provides an important contextual background within which the qualitative manifestations of motivational withdrawal were observed. Assessment literature often assumes that clearer assessment demands can activate student learning behavior (Fischer et al., 2024). In principle, this assumption is understandable: if students know what will be tested, they may be more likely to prepare. In the present data, however, some students described knowing the examination priorities while continuing to demonstrate limited review, passive attendance, or minimal engagement with learning materials.

The present study was not designed to determine whether clear examination guidance loses its motivating function or whether exam-oriented practices cause motivational withdrawal. Rather, the qualitative findings illustrate how diminished motivational support and perceived effort ineffectiveness were manifested during examination preparation and review activities. In these accounts, examination information remained visible, but students frequently described limited ownership of learning and little confidence that further effort would improve their outcomes.

This contextual pattern is consistent with SDT-based research showing that externally regulated demands are unlikely to sustain meaningful engagement when autonomous motivation is weak (Reeve and Cheon, 2021). The qualitative findings also illustrate the co-occurrence of learning experienced as externally required and beliefs that effort is unlikely to be effective. Pedagogically, the findings suggest that clear examination guidance alone may be insufficient to re-engage students who already experience diminished motivational support and perceived effort ineffectiveness.

### 5.5 Vocational context and implications

Finally, the vocational context is central to the situated interpretation of motivational withdrawal. As noted in the introduction and literature review, vocational college students often enter English classrooms with weak academic foundations, fragile confidence, and histories of educational marginalization (Gu and Schweisfurth, 2006). The qualitative findings indicate that this background was relevant to how participants understood English learning and their own capacity to improve. For many students, English was experienced not simply as difficult, but as irrelevant to future life, detached from realistic success, and repeatedly associated with failure. Some accounts further suggested that exam-oriented review was experienced less as meaningful learning support than as another formal requirement or reminder of difficult learning demands.

Pedagogically, the findings suggest that examination reminders alone may be insufficient to restore engagement unless teachers also rebuild task meaning, create achievable experiences of success, and address students’ limited confidence in the effectiveness of effort. This implication does not require the conclusion that examination practices cause motivational withdrawal. Instead, it highlights the need to respond to the two motivational dimensions identified in the present study within existing vocational English-learning contexts.

Meanwhile, the study should be read within its contextual and methodological limits. The sample came from three Chinese higher vocational colleges, while classroom observation was concentrated in one site. Although the present study employed an independent validation sample for CFA, the scale remains context-sensitive and requires broader validation across more diverse institutional settings. In addition, exam-oriented instructional practices were not independently measured, and the cross-sectional design cannot establish whether such practices contributed causally to motivational withdrawal or explain the mechanisms through which examination incentives may lose their motivating function. The present findings also do not suggest that all forms of disengagement observed in vocational English classrooms necessarily represent enduring motivational withdrawal. Some patterns may reflect temporary examination fatigue, low perceived utility value, selective disengagement from English specifically, or context-dependent responses associated with institutional assessment practices.

Even so, the study contributes a clearer account of the two-dimensional motivational structure underlying disengagement and demonstrates how diminished self-determined motivational support and perceived effort ineffectiveness were manifested in students’ reported experiences and observable engagement patterns. It therefore supports the conceptualization of disengagement in the present vocational English-learning context as a patterned form of motivational withdrawal rather than mere unwillingness.

## 6 Conclusion

This study examined the latent motivational structure and qualitative manifestations underlying vocational college students’ disengagement from English learning within exam-oriented instructional contexts. The qualitative findings showed that motivational withdrawal was manifested in recurrent non-engagement with review activities, including limited response to highlighted examination content and preparation guidance. More specifically, two closely related yet distinguishable dimensions were identified: diminished self-determined motivational support and perceived effort ineffectiveness. The proposed two-dimensional structure was supported through exploratory and confirmatory factor analyses conducted on separate samples, while the qualitative evidence illustrated how these dimensions were manifested in students’ reported experiences and observable engagement patterns.

From the perspectives of SDT and LHT, these findings suggest that students’ disengagement may reflect more than a temporary loss of interest or weak discipline. Diminished motivational support was manifested in students’ limited sense of ownership, agency, competence, and personal meaning, whereas perceived effort ineffectiveness was reflected in beliefs that further study or review was unlikely to improve learning outcomes. Together, these two dimensions provide an empirically grounded and context-sensitive account of motivational withdrawal among vocational English learners.

Pedagogically, the findings suggest that stronger examination reminders alone may be insufficient to re-engage students experiencing diminished motivational support and perceived effort ineffectiveness. Restoring engagement may also require teachers to strengthen task meaning, provide achievable experiences of success, and rebuild students’ confidence in the effectiveness of effort.

While the findings provide a context-sensitive explanation of motivational withdrawal in vocational English learning, the thematic construction remains inherently interpretive. Although the final thematic structure was judged to provide the most coherent interpretation across the available qualitative evidence, alternative readings of some individual participant accounts remain possible and should be acknowledged as a limitation of the present analytic framework. This study should be interpreted in light of its contextual and methodological boundaries, including its focus on three Chinese higher vocational colleges and its cross-sectional design. Because exam-oriented instructional practices were not independently measured, the findings should not be interpreted as establishing the mechanisms through which examination incentives may lose their motivating function. Within these boundaries, the study contributes a clearer account of the two-dimensional structure of motivational withdrawal and its qualitative manifestations in vocational English learning.

## Data Availability

The datasets generated during the current study are not publicly available because the qualitative data include classroom observations, platform behavioral records, and interview materials that may contain institutionally sensitive information and potentially identifiable participant details. Access to portions of the anonymized data may be considered by the corresponding author upon reasonable request and with institutional permission. Requests to access the datasets should be directed to 1315329804@qq.com.
