# Supplementary material for: Motivational withdrawal in college English learning: a mixed-methods investigation of diminished motivational support and perceived effort ineffectiveness
Source: Front Psychol. 2026 Aug 3;17:1872522. doi: 10.3389/fpsyg.2026.1872522 (PMC13478254; doi:10.3389/fpsyg.2026.1872522)
Supplement: Supplementary file 1 [file Data_Sheet_1.docx]

Appendix A. Questionnaire Items Used in the Study

The questionnaire consisted of 10 items measured on a five-point Likert scale ranging from 1 (strongly disagree) to 5 (strongly agree). The instrument included two theoretically informed dimensions: self-determined motivational support (SDT) and learned helplessness tendencies (LHT).

For the SDT dimension, higher scores indicated stronger self-determined motivational support toward English learning.

**For the LHT dimension, items were negatively worded and reverse-coded during statistical analysis. After reverse coding, higher scores represented lower levels of learned helplessness and stronger perceived motivational control.**

Table A1. English Version of Questionnaire Items

| **Code** | **Item** | **Dimension** | **Reverse-coded** |
| --- | --- | --- | --- |
| SDT1 | I believe learning English here in the university is meaningful for my future development. | Self-determined motivational support | No |
| SDT2 | I feel that I actively choose to engage in English learning activities. | Self-determined motivational support | No |
| SDT3 | I feel a sense of achievement when I complete English learning tasks. | Self-determined motivational support | No |
| SDT4 | I am willing to participate in English exam review activities. | Self-determined motivational support | No |
| SDT5 | I think English learning is worth investing time and effort in. | Self-determined motivational support | No |
| LHT1R | No matter how hard I study English, I still cannot improve my performance. | Learned helplessness tendency | Yes |
| LHT2R | Even when teachers provide key exam content, I still feel unable to do well. | Learned helplessness tendency | Yes |
| LHT3R | I often feel that reviewing English is useless because I will probably fail anyway. | Learned helplessness tendency | Yes |
| LHT4R | When I encounter difficulties in English learning, I usually feel that I cannot overcome them. | Learned helplessness tendency | Yes |
| LHT5R | Past failures in English learning make me believe that future effort will not help much. | Learned helplessness tendency | Yes |

Table A2. Chinese Version of Questionnaire Items

| **编号** | **中文题项** | **维度** | **是否反向计分** |
| --- | --- | --- | --- |
| SDT1 | 我认为在大学这儿学习英语对我未来的发展是有意义的。 | 自主动机支持 | 否 |
| SDT2 | 我觉得自己是主动选择参与英语学习活动的。 | 自主动机支持 | 否 |
| SDT3 | 当我完成英语学习任务时，我会感到一种成就感。 | 自主动机支持 | 否 |
| SDT4 | 我愿意参与英语考试复习活动。 | 自主动机支持 | 否 |
| SDT5 | 我认为英语学习值得投入时间和精力。 | 自主动机支持 | 否 |
| LHT1R | 无论我多么努力学习英语，我仍然无法提高自己的成绩。 | 习得性无助倾向 | 是 |
| LHT2R | 即使老师提供了考试重点内容，我仍然觉得自己无法学好。 | 习得性无助倾向 | 是 |
| LHT3R | 我经常觉得复习英语是没有用的，因为我最终可能还是会失败。 | 习得性无助倾向 | 是 |
| LHT4R | 当我在英语学习中遇到困难时，我通常觉得自己无法克服它们。 | 习得性无助倾向 | 是 |
| LHT5R | 过去英语学习中的失败经历让我觉得未来再努力也没有太大帮助。 | 习得性无助倾向 | 是 |
