# Supplementary material for: Motivational withdrawal in college English learning: a mixed-methods investigation of diminished motivational support and perceived effort ineffectiveness
Source: Front Psychol. 2026 Aug 3;17:1872522. doi: 10.3389/fpsyg.2026.1872522 (PMC13478254; doi:10.3389/fpsyg.2026.1872522)
Supplement: Supplementary file 2 [file Data_Sheet_2.docx]

# Open-Ended Questionnaire

## Open-Ended Questionnaire for Vocational College Students’ English Learning Experiences

Dear student,

This questionnaire aims to understand vocational college students’ experiences, attitudes, and perceptions regarding English learning in exam-oriented educational contexts. There are no right or wrong answers. Please answer honestly based on your own learning experiences. All responses will be kept confidential and used only for academic research purposes.

## Section A: English Learning Experience

### Q1. How do you usually feel about learning English in college? Please describe your general attitudes and experiences.

### Q2.What aspects of English learning do you find most difficult or frustrating? Why?

### Q3. Can you describe any experiences in which you felt unsuccessful, discouraged, or disappointed in English learning?

### Q4. Do you think English learning is meaningful or useful for your future life, work, or personal development? Why or why not?

## Section B: Exam-Oriented Learning and Review Behavior

### Q5. How do you usually prepare for English exams? Please describe your review habits and behaviors.

### Q6. Teachers often provide important review materials, key vocabulary, or predicted exam content before exams. How do you usually respond to these review activities?

### Q7. Have you ever known that an exam was important but still felt unwilling to review or prepare for it? Please explain your thoughts and feelings in such situations.

## Section C: Motivation and Effort Perceptions

### Q8. When you study English seriously but still achieve unsatisfactory results, how do you usually feel?

### Q9. Do you believe that making more effort in English learning will eventually lead to improvement? Why or why not?

### Q10. Have you ever felt that no matter how hard you study English, the result will not change very much? Please describe your experience.

### Q11. What factors most influence your motivation to learn English?

## Section D: Classroom Participation and Engagement

### Q12. How do you usually participate in English classes?

### Q13. What kinds of classroom activities make you more willing to participate in English learning?

### Q14. What kinds of classroom situations make you lose motivation or stop participating?

### Section E: Suggestions and Reflections

### Q15. What do you think teachers could do to make English learning more meaningful or motivating for vocational college students?

### Q16. If you could change one thing about College English courses or exam systems, what would you change? Why?

**(Original Chinese version)**

**开放式问卷**

**高职院校学生英语学习经历开放式问卷**

亲爱的同学：

本问卷旨在了解高职院校学生在应试教育背景下的英语学习经历、态度与感受。问卷没有对错之分，请根据你自己的真实学习经历如实回答。所有回答仅用于学术研究，并将严格保密。

**第一部分：英语学习经历**

Q1. 你通常如何看待大学里的英语学习？请描述你对英语学习的总体态度和经历。

Q2. 你认为英语学习中哪些方面最困难或最让你感到挫败？为什么？

Q3. 你能描述一些在英语学习中感到失败、沮丧或失望的经历吗？

Q4. 你认为英语学习对你未来的生活、工作或个人发展有意义或有用吗？为什么或为什么不？

**第二部分：应试学习与复习行为**

Q5. 你通常如何准备英语考试？请描述你的复习习惯和行为。

Q6. 老师通常会在考试前提供重点复习资料、关键词汇或预测考试内容。你通常如何对待这些复习活动？

Q7. 你是否曾经明知道考试很重要，但仍然不愿意复习或准备？请说明当时的想法和感受。

**第三部分：动机与努力认知**

Q8. 当你认真学习英语却依然取得不理想的成绩时，你通常会有什么感受？

Q9. 你是否相信在英语学习中付出更多努力最终会带来进步？为什么或为什么不？

Q10. 你是否曾经觉得无论自己多么努力学习英语，结果都不会有太大改变？请描述你的经历。

Q11. 哪些因素最能影响你学习英语的动力？

**第四部分：课堂参与与投入**

Q12. 你通常如何参与英语课堂？

Q13. 哪些类型的课堂活动会让你更愿意参与英语学习？

Q14. 哪些课堂情境会让你失去学习动力或停止参与？

**第五部分：建议与反思**

Q15. 你认为老师可以做些什么，让高职院校学生觉得英语学习更有意义或更有动力？

Q16. 如果你可以改变大学英语课程或考试制度中的一件事，你会改变什么？为什么？
