# Supplementary material for: Motivational withdrawal in college English learning: a mixed-methods investigation of diminished motivational support and perceived effort ineffectiveness
Source: Front Psychol. 2026 Aug 3;17:1872522. doi: 10.3389/fpsyg.2026.1872522 (PMC13478254; doi:10.3389/fpsyg.2026.1872522)
Supplement: Supplementary file 3 [file Data_Sheet_3.docx]

## Focus Group Interview Guide

### Introduction

Thank you for participating in this interview.
This discussion aims to understand vocational college students’ experiences and feelings about English learning, exam preparation, and classroom participation. There are no right or wrong answers. Please speak freely based on your own experiences. All information will be kept confidential and used only for academic research purposes.

## Interview Questions

### 1.Based on your own experience, how would you describe your experience of learning English in vocational college? Could you give some examples?

### 2. Can you describe your experiences of preparing for English exams in vocational college?

### 3. Teachers often provide important review materials or key exam content before exams. How do you students usually respond to these review activities?

### 4. Have you ever experienced a situation in which you did not prepare for an English exam as much as you had originally intended? If so, could you describe what happened and what influenced your decision?

### 5. What kinds of experiences do you think help or hinder students’ progress in English learning?

### 6. What kinds of classroom activities change your willingness to participate in English learning?

### 7. What kinds of factors outside the classroom, if any, influence students’ willingness to participate in English learning?

### 8. In your opinion, why do different students participate in English learning in different ways?

### 9. What could teachers or schools do to make English learning more meaningful or motivating for students?

## Closing

Thank you for your participation and sharing your experiences. Your responses are very valuable for this research.

**（Original Chinese version)**

焦点小组访谈提纲

引言

感谢你参与本次访谈。

本次讨论旨在了解高职院校学生对于英语学习、考试准备以及课堂参与的经历和感受。这里没有正确或错误的答案，请根据您自己的真实经历自由表达。所有信息都将严格保密，仅用于学术研究目的。

访谈问题

1. 根据你自己的经历，你会如何描述自己在高职院校学习英语的体验？你能举一些例子吗？

2. 你能描述一下你在高职院校准备英语考试的经历吗？

3. 老师通常会在考试前提供重要的复习资料或考试重点内容。对于这些复习活动，学生们通常会有什么样的反应？

4. 你是否曾经历过这样一种情况：你没有像自己原本计划的那样充分准备英语考试？如果有，你能描述一下当时发生了什么，以及哪些因素影响了你的决定吗？

5. 你认为哪些类型的经历会帮助或阻碍学生在英语学习中的进步？

6. 哪些类型的课堂活动会改变你参与英语学习的意愿？

7. 哪些课堂之外的因素（如果有的话）会影响学生参与英语学习的意愿？

8. 你认为为什么不同的学生会以不同的方式参与英语学习？

9. 你认为教师或学校可以采取哪些措施，使英语学习对学生来说更有意义或更具激励作用？

结束语

感谢你的参与和经历分享。你的回答对本研究非常有价值。
