# Supplementary material for: Motivational withdrawal in college English learning: a mixed-methods investigation of diminished motivational support and perceived effort ineffectiveness
Source: Front Psychol. 2026 Aug 3;17:1872522. doi: 10.3389/fpsyg.2026.1872522 (PMC13478254; doi:10.3389/fpsyg.2026.1872522)
Supplement: Supplementary file 4 [file Data_Sheet_4.docx]

Teacher Interview Guide

**Introduction**

Thank you for participating in this interview.

This interview aims to explore teachers’ observations and interpretations of vocational college students’ English learning motivation, exam preparation behaviors, and classroom engagement in exam-oriented instructional contexts. All information will be kept confidential and used only for academic research purposes.

**Interview Questions**

**1.How would you describe your students’ overall attitudes toward College English learning?**

2. **What changes have you observed in students’ exam preparation behaviors in recent years?**
3. **Teachers often provide important review materials, key language points, or predicted exam content before exams. In your experience, how do students usually respond to these review activities?**
4. **Can you recall a situation in which an English exam was particularly important? How did students usually prepare, and what different patterns of preparation did you observe?**
5. **In your experience, how do different students respond when explicit exam requirements or review guidance are provided? What factors do you think influence these different responses?**
6. **Based on your classroom experience, what factors seem to influence how students respond to exam preparation and classroom learning?**
7. **What kinds of classroom behaviors or participation patterns have you observed among students during English classes? Which of these seem particularly meaningful to you, and why?**
8. **Can you describe how you usually respond when students participate in English learning or exam preparation in different ways?**
9. **In your experience, what factors seem to influence students’ participation and learning in College English?**
10. **What changes do you think could improve students’ motivation and participation in vocational English classrooms?**

(Original Chinese version)

教师访谈指南

**引言**

感谢您参与本次访谈。

本次访谈旨在探讨教师对职业院校学生英语学习动机、考试准备行为以及在应试导向教学情境中的课堂参与情况的观察与理解。所有信息将严格保密，仅用于学术研究目的。

**访谈问题**

1. 您会如何描述学生对大学英语学习的整体态度？

2. 近年来，您观察到学生在英语考试备考行为方面发生了哪些变化？

3. 教师通常会在考试前提供重要的复习资料、语言重点或预测考试内容。根据您的经验，学生通常会如何回应这些复习活动？

4. 您能回忆一次英语考试对学生而言特别重要的情况吗？学生通常是如何备考的？您观察到了哪些不同的备考模式？

5. 根据您的经验，当教师提供明确的考试要求或复习指导时，不同学生通常会作出怎样的反应？您认为哪些因素会影响这些不同的反应？

6. 根据您的课堂教学经验，哪些因素似乎会影响学生对考试备考和课堂学习的反应？

7. 在英语课堂中，您观察到学生有哪些课堂行为或课堂参与模式？其中哪些对您来说尤其有意义？为什么？

8. 您通常会如何回应学生在英语学习或考试备考过程中表现出的不同参与方式？

9. 根据您的经验，哪些因素似乎会影响学生在大学英语学习中的课堂参与和学习表现？

10. 您认为哪些改变可以提高高职英语课堂中学生的学习动机和课堂参与度？
