# Supplementary material for: Motivational withdrawal in college English learning: a mixed-methods investigation of diminished motivational support and perceived effort ineffectiveness
Source: Front Psychol. 2026 Aug 3;17:1872522. doi: 10.3389/fpsyg.2026.1872522 (PMC13478254; doi:10.3389/fpsyg.2026.1872522)
Supplement: Supplementary file 5 [file Data_Sheet_5.docx]

**Appendix E
Coding Process, Code Refinement, and Theme Development**

1. Data preparation

All qualitative data were collected from multiple sources, including open-ended questionnaire responses, focus group interview transcripts, classroom observation records, platform observation records, and teacher interviews. The qualitative dataset consisted of 194 valid open-ended questionnaire responses (SQ1–SQ194), one focus group interview with ten student participants (SI1–SI10), ten classroom observation records (CO1–CO10), platform observation records generated during the pre-exam period, and five teacher interviews (TI1–TI5).

All interview recordings were transcribed verbatim before analysis. The open-ended questionnaire responses, student interview transcripts, classroom observation notes, platform records, and teacher interview transcripts were imported into NVivo 14 for data organization and coding. During the preparation stage, the researcher repeatedly read the full dataset to develop familiarity with participants’ accounts, recurring behavioral patterns, and contextual meanings related to exam-oriented English learning. Particular attention was paid to how students explained their attitudes toward English, their review behavior, their perceptions of exam preparation, and their beliefs about whether effort could improve English performance.

Because the study adopted a theory-informed thematic analysis guided by Self-Determination Theory (SDT) and Learned Helplessness Theory (LHT), the analysis was not conducted as a purely inductive process. However, SDT and LHT were treated as sensitizing concepts rather than as rigid coding categories. This means that the researcher used concepts such as autonomy, competence, task meaning, perceived control, failure expectation, and effort ineffectiveness to guide attention during analysis, while also remaining open to meanings that emerged from participants’ own descriptions and classroom behavior.

2. Initial coding process

The first coding cycle combined deductive and inductive coding. Deductive coding was informed by the theoretical framework of the study, especially the SDT-related concepts of autonomy, competence, task value, and learning ownership, and the LHT-related concepts of repeated failure, perceived effort ineffectiveness, anticipated failure, and withdrawal. Inductive coding was used when participants’ responses or observed classroom behaviors reflected meanings that were not fully captured by the initial theoretical framework.

During the first coding cycle, the researcher identified data segments related to weak task meaning, externally controlled learning, passive classroom engagement, limited willingness to participate in review activities, anticipated failure, perceived effort ineffectiveness, and disengagement despite continuing examination pressure or instructional support. Initial codes were attached to short units of meaning, including sentences, clauses, short questionnaire responses, interview extracts, and observation notes.

Table E1 provides representative examples of the transformation from raw extracts or observation records into initial codes. These initial codes formed the basis for subsequent code refinement, category development, and theme construction. The later tables document how codes were consolidated into broader categories, linked to final themes, supported across multiple data sources, and examined in relation to their relative recurrence within the dataset.

**Table E1. Representative raw-data-to-code examples**

| Data source | Representative raw extract or observation record (examples) | Initial code |
| --- | --- | --- |
| SQ127 | “I’m not going abroad, so English is useless to me.” | Lack of future relevance |
| SQ41 | “No one in my family speaks English, and no one requires me to learn it.” | Weak social relevance of English |
| SQ8 | “English review is something completed only because it was required before exams.” | Externally imposed review |
| SQ26 | “Review materials were not personally meaningful and were mainly treated as exam requirements.” | Review as routine obligation |
| SI9 | “I only attend English classes because the teacher is engaging, not because I want to learn English.” | Attendance without learning ownership |
| SI7 | “I want to sleep, and play games. I feel depressed and bored here, but I have to stay here.” | Passive classroom presence |
| SQ88 | “I think I reviewed, but I don’t think I will succeed” | Reviewing with anticipated failure |
| SI6 | “Effort in English is actually useless because previous effort had not produced visible improvement.” | Perceived uselessness of effort |
| CO1 | Some students remained seated but did not open textbooks or take notes during English instruction. | Passive attendance |
| CO5 | Several students used mobile phones or chatted while the teacher explained review content. | Non-engagement during review |
| CO7 | Some students ignored review materials even after the teacher highlighted likely exam content. | Ignoring review materials |
| CO9 | A student responded to the teacher’s explanation by saying, “You can keep explaining, but I won’t get it.” | Anticipated inability to understand |
| CO9–CO10 | Platform records showed low submission rates and limited interaction with review materials during the final pre-exam period. | Low online review activity |
| TI2 | “Exam reminders did not always lead students to begin review.” | Non-engagement during exam-oriented review |
| TI4 | “Some students no longer believed concentrating on key exam content would make a difference.” | Low confidence in review effectiveness |
| TI5 | “Some students’ responses to review support are passive and they think preparation was pointless.” | Preparation perceived as pointless |

These initial codes remained provisional. They were not treated as final categories. Instead, the researcher returned to the dataset repeatedly to compare similar data segments across questionnaire responses, interview accounts, classroom observations, platform records, and teacher interviews.

3. Iterative code refinement

After the first coding cycle, codes were compared across data sources to identify overlap, clarify boundaries, and refine conceptual meanings. Similar codes were merged into broader analytic categories, whereas conceptually distinct codes were retained as separate categories.

For example, codes such as “English is useless,” “lack of future relevance,” and “weak social relevance of English” were grouped under weak task meaning because they reflected students’ limited perception of English as personally, socially, or occupationally meaningful. These responses were interpreted primarily as indicators of diminished task value rather than direct evidence of learned helplessness.

Similarly, codes associated with anticipated failure, perceived ineffectiveness of effort, and disengagement despite examination pressure were refined into broader categories that captured different aspects of students’ motivational withdrawal. Table E2 shows how early codes were consolidated into broader analytic categories through this refinement process.

**Table E2. Code refinement and category development**

| **Early codes** | **Refined category** | **Analytic interpretation** |
| --- | --- | --- |
| Lack of future relevance; Weak social relevance of English | Weak task meaning | English was perceived as lacking personal, social, or occupational relevance, reducing students’ willingness to invest effort in learning. |
| Externally imposed review; Review as routine obligation; Attendance without learning ownership | Externally controlled learning | Learning participation was sustained primarily through institutional requirements and examination demands rather than internal endorsement or ownership. |
| Passive classroom presence; Passive attendance; Non-engagement during review | Low classroom engagement | Students remained physically present but demonstrated limited cognitive, emotional, and behavioral involvement in classroom learning activities. |
| Ignoring review materials; Low online review activity | Reduced participation willingness | Students showed limited willingness to engage with teacher-provided learning opportunities and review resources even when these were readily available. |
| Reviewing with anticipated failure; Low confidence in review effectiveness | Failure expectation | Students anticipated unsuccessful outcomes and doubted whether preparation would lead to improvement or success. |
| Perceived uselessness of effort; Preparation perceived as pointless | Perceived effort ineffectiveness | Students believed that effort was unlikely to produce meaningful learning gains or improved examination performance. |
| Anticipated inability to understand; Disengagement despite exam pressure | Helplessness-related disengagement | Students perceived limited control over learning outcomes and remained disengaged even when explicit examination incentives or instructional support were available. |

This refinement process involved repeated movement between coded extracts, theoretical concepts, and the broader dataset. The researcher examined whether each category was supported by multiple data sources and whether it contributed to explaining how the two identified dimensions were manifested in students’ reported experiences and observable engagement patterns.

4. Considering alternative interpretations

During code refinement, the researcher did not assume that theoretically informed interpretations represented the only possible explanation of participants’ statements. When a data extract could reasonably support more than one interpretation, alternative explanations were explicitly compared before a final analytic decision was made.

For example, statements such as “English is useless to me” were first examined as possible evidence of occupational irrelevance, pragmatic instrumental beliefs, curriculum-context mismatch, or weak task meaning. The researcher then returned to the surrounding interview context, questionnaire responses, classroom observations, platform records, and teacher interviews to determine which interpretation was most consistently supported across the dataset.

A theoretically informed interpretation was retained only when it explained not only the focal quotation but also the broader motivational pattern evident across multiple qualitative sources. Where competing explanations remained equally plausible, the coding remained descriptive at the initial stage and theoretical interpretation was deferred until additional contextual evidence became available.

This iterative comparison reduced premature theoretical closure and helped ensure that final themes represented analytically justified interpretations rather than automatic applications of SDT or LHT concepts.

Table E3 shows illustrative comparison of alternative interpretations during code refinement.

**Table E3 Illustrative comparison of alternative interpretations during code refinement.**

| **Representative extract** | **Alternative interpretation(s) considered** | **Final interpretation retained** | **Basis for interpretive decision** |
| --- | --- | --- | --- |
| **"I'm not going abroad, so English is useless to me." (SQ127)** | Occupational irrelevance; pragmatic instrumental belief; weak task meaning | **Weak task meaning** | The statement was compared with surrounding questionnaire responses and interview accounts. The participant emphasised the limited personal value of English rather than curriculum design or employment requirements. Similar expressions recurred across multiple participants and were therefore interpreted as diminished task meaning. |
| **"English review is something completed only because it was required before exams." (SQ8)** | Examination compliance; externally imposed learning; curriculum-context mismatch | **Externally controlled learning** | The statement described participation driven by institutional requirements rather than personal endorsement. Similar patterns were supported by interview and classroom observation data showing review as obligation rather than self-directed learning. |
| **"I think I reviewed, but I don't think I will succeed." (SQ88)** | Low English proficiency; examination anxiety; failure expectation | **Failure expectation** | The participant explicitly linked review behaviour with anticipated failure rather than describing temporary anxiety or lack of ability alone. Comparable accounts appeared across interviews and teacher reports. |
| **"Effort in English is actually useless because previous effort had not produced visible improvement." (SI6)** | Low confidence; frustration with teaching; perceived effort ineffectiveness | **Perceived effort ineffectiveness** | The participant directly connected repeated effort with the absence of improvement. Similar explanations were repeatedly observed across questionnaires, interviews, and teacher accounts, indicating a broader perception that effort would not change learning outcomes. |
| **"You can keep explaining, but I won't get it." (CO9)** | Low language proficiency; temporary classroom disengagement; helplessness-related disengagement | **Helplessness-related disengagement** | This observation was interpreted together with questionnaire responses, classroom behaviour, and teacher interviews. The interpretation was retained because the behavioural pattern reflected disengagement despite continuing instructional support rather than an isolated episode of misunderstanding. |

5. Development from categories to themes

After code refinement, related categories were grouped into broader themes. This process was not a mechanical aggregation of codes. Rather, categories were reviewed in relation to the theoretical framework, the research questions, and the overall pattern of qualitative and quantitative findings.

Two final themes were developed.

Theme 1: Diminished self-determined motivational support

This theme captured students’ weakened sense of task meaning, learning ownership, and active engagement in English learning. It was developed from categories including weak task meaning, externally controlled learning, low classroom engagement, and reduced participation willingness. Together, these categories reflected students’ tendency to experience English learning as externally imposed, weakly connected to their personal goals, and lacking sufficient personal relevance.

Theme 2: Perceived effort ineffectiveness and failure expectation

This theme captured students’ belief that effort, review, and instructional support were unlikely to produce meaningful improvement in English-learning outcomes. It was developed from categories including failure expectation, perceived effort ineffectiveness, and helplessness-related disengagement. These categories captured recurring expressions of anticipated failure, limited confidence in the effectiveness of effort, and disengagement observed during examination-related activities.

**Table E4. Code-to-category-to-theme matrix**

| **Initial codes** | **Refined category** | **Final theme** |
| --- | --- | --- |
| Lack of future relevance; Weak social relevance of English | Weak task meaning | Diminished self-determined motivational support |
| Externally imposed review; Review as routine obligation; Attendance without learning ownership | Externally controlled learning | Diminished self-determined motivational support |
| Passive classroom presence; Passive attendance; Non-engagement during review | Low classroom engagement | Diminished self-determined motivational support |
| Ignoring review materials; Low online review activity | Reduced participation willingness | Diminished self-determined motivational support |
| Reviewing with anticipated failure; Low confidence in review effectiveness | Failure expectation | Perceived effort ineffectiveness and failure expectation |
| Perceived uselessness of effort; Preparation perceived as pointless | Perceived effort ineffectiveness | Perceived effort ineffectiveness and failure expectation |
| Anticipated inability to understand; Disengagement despite exam pressure | Helplessness-related disengagement | Perceived effort ineffectiveness and failure expectation |

6. Illustrative Analytic Trails

To enhance transparency in the analytic process, Table E5 presents representative analytic trails illustrating how selected data extracts were interpreted, coded, refined into analytic categories, and ultimately linked to the final themes.

**Table E5. Illustrative analytic trails from raw data to final themes (examples)**

| **Raw extract or observation record examples** | **Initial code** | **Refined category** | **Final theme** | **Interpretive reasoning** |
| --- | --- | --- | --- | --- |
| “I’m not going abroad, so English is useless to me.” (SQ127) | Lack of future relevance | Weak task meaning | Diminished self-determined motivational support | The student framed English as unrelated to future goals and therefore lacking personal value. The response was interpreted as reduced task meaning rather than evidence of helplessness. |
| “I only attend English classes because the teacher is engaging, not because I want to learn English.” (SI9) | Attendance without learning ownership | Externally controlled learning | Diminished self-determined motivational support | Participation was maintained by external classroom conditions rather than internal endorsement of learning. |
| “I want to sleep and play games. I feel depressed and bored here, but I have to stay here.” (SI7) | Passive classroom presence | Low classroom engagement | Diminished self-determined motivational support | The student was physically present but psychologically disengaged, indicating limited active involvement in learning. |
| Some students ignored review materials even after the teacher highlighted likely exam content. (CO7) | Ignoring review materials | Reduced participation willingness | Diminished self-determined motivational support | Students chose not to engage with available review opportunities despite explicit guidance and exam relevance. |
| “I think I reviewed, but I don’t think I will succeed.” (SQ88) | Reviewing with anticipated failure | Failure expectation | Perceived effort ineffectiveness and failure expectation | Preparation was accompanied by an expectation of failure, suggesting low confidence in the effectiveness of review. |
| “Effort in English is actually useless because previous effort had not produced visible improvement.” (SI6) | Perceived uselessness of effort | Perceived effort ineffectiveness | Perceived effort ineffectiveness and failure expectation | The student connected current disengagement to repeated experiences in which effort appeared ineffective. |
| “You can keep explaining, but I won’t get it.” (CO9) | Anticipated inability to understand | Helplessness-related disengagement | Perceived effort ineffectiveness and failure expectation | The statement reflected a perceived lack of control over learning outcomes and disengagement despite continuing instructional support. |

These analytic trails show how the final themes were derived through repeated movement between raw extracts, initial codes, refined categories, and theoretical interpretation. They also demonstrate that the two themes were analytically related but conceptually distinct. Theme 1 primarily concerned students’ diminished sense of task meaning, learning ownership, and active engagement in English learning, whereas Theme 2 concerned students’ perceptions that effort, review, and instructional support were unlikely to improve learning outcomes; these perceptions constituted one qualitative manifestation of motivational withdrawal.

7. Cross-data triangulation

To enhance analytical trustworthiness, emerging codes and themes were repeatedly compared across different qualitative sources. The purpose of triangulation was not to force all data sources into identical conclusions, but to examine whether similar motivational patterns appeared across different forms of evidence. Table E6 summarizes how the major themes were supported across data sources.

**Table E6. Cross-data triangulation of final themes**

| **Data source** | **Evidence for diminished self-determined motivational support** | **Evidence for Perceived effort ineffectiveness and failure expectation** |
| --- | --- | --- |
| Open-ended questionnaires | Students described English as meaningless, irrelevant to future plans, or disconnected from daily life. | Students reported that review was unlikely to improve outcomes and that previous effort had not produced visible progress. |
| Focus group interview | Students described attending English classes without wanting to learn English, or participating because of classroom requirements rather than personal interest. | Students described effort as useless, review as ineffective, or failure as likely even when they understood that exams were important. |
| Classroom observations | Students were physically present but often did not open textbooks, take notes, or respond to teacher guidance. Some used mobile phones, chatted, or rested during review. | Students remained passive even during pre-exam review sessions and sometimes expressed that they would not understand even if the teacher continued explaining. |
| Platform observation | Some students maintained nominal participation in the online learning system but showed limited actual engagement with review materials. | Low submission rates and limited interaction with review resources continued during the final pre-exam period. |
| Teacher interviews | Teachers reported that many students treated English as something to endure rather than something to engage with. | Teachers reported that some students no longer believed review or concentration on key exam content would make a difference. |

The triangulation process strengthened the credibility of the two final themes. The first theme was supported by students’ stated lack of task meaning, observed passive participation, and teachers’ reports of externally sustained classroom attendance. The second theme was supported by students’ reports of anticipated failure and effort ineffectiveness, observations of disengagement despite instructional support, low platform activity during the pre-exam period, and teachers’ reports that some students no longer believed review could improve outcomes.

8. Negative Cases and Contextual Variation

The analysis also considered responses that did not fully align with the dominant interpretation of motivational withdrawal. Not all disengagement was interpreted as direct evidence of learned helplessness or motivational withdrawal. Some students appeared to evaluate English pragmatically in relation to future employment, occupational relevance, transfer examinations, certificate requirements, or immediate life priorities. For example, several questionnaire responses suggested that English was viewed as having limited relevance to anticipated occupational trajectories rather than as a subject in which effort was necessarily perceived as ineffective.

These cases were important because they suggested that not all forms of disengagement reflected perceived effort ineffectiveness or helplessness-related beliefs. In some instances, disengagement appeared to arise from low perceived task value, limited future relevance, or pragmatic evaluations of English-learning usefulness. Such responses were therefore treated primarily as evidence of weak task meaning rather than direct evidence of helplessness-related disengagement.

Similarly, teacher interviews indicated that a small number of students continued to demonstrate instrumental or achievement-oriented motivation. These students were typically described as maintaining engagement when English learning was connected to transfer examinations, certificate acquisition, or specific employment-related goals. Compared with other participants, they were more likely to participate in review activities and maintain a clearer sense of learning purpose.

These negative and variant cases were retained throughout coding and theme development rather than excluded from analysis. Their presence contributed to the refinement of thematic boundaries and helped distinguish diminished task meaning from perceived effort ineffectiveness.

Therefore, motivational withdrawal was interpreted as a prominent pattern within the present dataset rather than a universal condition among all vocational college students. The final themes were developed to explain recurring tendencies across the dataset while still allowing for contextual variation, selective engagement, and pragmatic responses. Alternative interpretations, including occupational irrelevance, curriculum-context mismatch, and pragmatic instrumental beliefs, were retained as contextual explanations where appropriate but were not elevated to final themes because they occurred less consistently across data sources and did not account for the broader motivational patterns evident throughout the dataset.

9. Reflexivity and Theoretical Sensitivity

Throughout the analytic process, reflexive memos were used to document analytic decisions, emerging interpretations, researcher assumptions, and potential interpretive bias. Because the study was guided by SDT and LHT, the researcher paid particular attention to the risk of over-interpreting all disengagement as evidence of motivational withdrawal. To reduce this risk, coded extracts were repeatedly compared with their surrounding context, and alternative explanations were considered.

For example, when students described English as useless for future employment, such responses were not automatically coded as learned helplessness. They were first examined as possible evidence of weak task meaning, pragmatic evaluation, or low perceived future relevance. Only when responses also included statements about effort being useless, improvement being unlikely, failure being expected, or disengagement despite continuing instructional support were they interpreted as evidence of perceived effort ineffectiveness and failure expectation.

Similarly, classroom non-engagement was not treated as sufficient evidence of motivational withdrawal by itself. Behaviors such as phone use, lack of note-taking, or passive attendance were interpreted alongside questionnaire responses, interview accounts, platform records, and teacher reports. This cross-checking helped avoid relying on isolated observations and reduced the risk of researcher overinterpretation.

The interpretive pathway presented in Tables E1–E5 was used to document how coding decisions evolved from raw extracts to final themes. Formal intercoder reliability procedures were not employed because the study followed an interpretivist and reflexive thematic analysis orientation. Coding was understood as a reflexive and theoretically informed analytic process rather than a procedure for producing statistically measurable agreement. Nevertheless, coding consistency was strengthened through repeated data immersion, iterative code refinement, comparison across multiple data sources, and revisiting portions of the coded dataset at different stages of analysis. Selected coding interpretations and thematic boundaries were also discussed with an experienced qualitative research colleague familiar with vocational English education.

10. Final Thematic Structure

The final thematic structure integrated both SDT and LHT perspectives and resulted in a two-dimensional representation of motivational withdrawal.

**Table E7. Final thematic structure**

| **Final theme** | **Core interpretation** | **Main categories** | **Representative evidence** | **Theoretical relevance** |
| --- | --- | --- | --- | --- |
| **Diminished self-determined motivational support** | Students’ English learning was weakened by low task meaning, limited learning ownership, externally controlled participation, and reduced active engagement. English was often experienced as something required by examinations rather than personally endorsed as meaningful or useful. | Weak task meaning; Externally controlled learning; Low classroom engagement; Reduced participation willingness | Students described English as unrelated to future goals or social needs; some attended English classes without wanting to learn English; classroom observations showed passive presence, lack of note-taking, mobile phone use, and limited engagement with review materials; platform records showed weak interaction with review resources. | This theme corresponds mainly to the SDT-informed dimension of the analysis. It reflects weakened task value, limited autonomy, reduced ownership, and low engagement within exam-oriented vocational English-learning contexts. |
| **Perceived effort ineffectiveness and failure expectation** | Students’ disengagement was associated with the belief that effort, review, or teacher guidance would not meaningfully improve English-learning outcomes. Accounts of repeated difficulty and limited visible improvement were accompanied by anticipated failure, low confidence in review effectiveness, and disengagement despite continuing examination pressure. | Failure expectation; Perceived effort ineffectiveness; Helplessness-related disengagement | Students reported reviewing while expecting failure, described English effort as useless, or regarded preparation as pointless; some expressed that they would not understand even if the teacher continued explaining; teachers reported that some students no longer believed concentration on key exam content would make a difference; low platform activity continued during the pre-exam period. | This theme corresponds mainly to the LHT-informed dimension of the analysis. It reflects perceived lack of control, anticipated failure, and effort-nullifying beliefs associated with motivational withdrawal within contexts where explicit exam guidance was present. |

Together, these themes provide an interpretive account of the two-dimensional structure of motivational withdrawal and its qualitative manifestations among vocational English learners. Examination preparation, teacher reminders, and exam-related review activities are treated as contextual settings in which these patterns became visible, rather than as independently measured causes of motivational withdrawal. The findings are context-specific and do not establish a causal mechanism through which exam-oriented instruction produces motivational withdrawal.

11. Code Frequency and Theme Coverage

Although reflexive thematic analysis does not treat code frequency as a direct indicator of analytical importance, the researcher recorded the relative recurrence of major codes across the qualitative dataset to improve transparency. The purpose of Table E8 is not to quantify themes statistically, but to demonstrate that the final thematic structure was supported by repeated patterns identified across multiple participants, observations, and data sources.

**Table E8 Code frequency and theme coverage**

| **Initial code** | **Refined category** | **Relative frequency** | **Main supporting data sources** |
| --- | --- | --- | --- |
| Lack of future relevance | Weak task meaning | High | SQ, SI |
| Weak social relevance of English | Weak task meaning | Moderate | SQ |
| Externally imposed review | Externally controlled learning | Moderate | SQ, SI |
| Review as routine obligation | Externally controlled learning | Moderate | SQ, SI |
| Attendance without learning ownership | Externally controlled learning | Moderate | SI, CO |
| Passive classroom presence | Low classroom engagement | High | SI, CO |
| Passive attendance | Low classroom engagement | Moderate | CO |
| Non-engagement during review | Low classroom engagement | High | CO |
| Ignoring review materials | Reduced participation willingness | High | SI, SQ, CO |
| Low online review activity | Reduced participation willingness | Moderate | Platform Observation |
| Reviewing with anticipated failure | Failure expectation | High | SQ, SI |
| Low confidence in review effectiveness | Failure expectation | Moderate | TI |
| Perceived uselessness of effort | Perceived effort ineffectiveness | High | SQ, SI |
| Preparation perceived as pointless | Perceived effort ineffectiveness | Moderate | TI |
| Anticipated inability to understand | Helplessness-related disengagement | Moderate | CO |
| Disengagement despite exam pressure | Helplessness-related disengagement | Moderate | TI, CO |

Relative frequency indicates the recurrence of codes across the qualitative dataset rather than precise occurrence counts. High = recurrent across multiple participants and data sources; Moderate = repeatedly identified but less pervasive; Low = limited but analytically important occurrence.

**12 Theme Coverage Matrix**

To further demonstrate analytical trustworthiness, Table E9 maps the relationship between the refined categories and the qualitative data sources from which supporting evidence was obtained. The purpose of this matrix is to show how thematic development was informed by converging evidence across multiple forms of qualitative data rather than relying on a single source of interpretation.

**Table E9. Theme Coverage Matrix**

| **Refined category** | **Open-ended questionnaire (SQ)** | **Student interview (SI)** | **Classroom observation (CO)** | **Platform observation** | **Teacher interview (TI)** |
| --- | --- | --- | --- | --- | --- |
| Weak task meaning | ✓ | ✓ |  |  |  |
| Externally controlled learning | ✓ | ✓ | ✓ |  | ✓ |
| Low classroom engagement |  | ✓ | ✓ |  | ✓ |
| Reduced participation willingness |  |  | ✓ | ✓ | ✓ |
| Failure expectation | ✓ | ✓ |  |  | ✓ |
| Perceived effort ineffectiveness | ✓ | ✓ |  |  | ✓ |
| Helplessness-related disengagement |  |  | ✓ | ✓ | ✓ |

Theme 1: Diminished self-determined motivational support

- Weak task meaning
- Externally controlled learning
- Low classroom engagement
- Reduced participation willingness

Theme 2: Perceived effort ineffectiveness and failure expectation

- Failure expectation
- Perceived effort ineffectiveness
- Helplessness-related disengagement

The coverage matrix indicates that each refined category was supported by more than one qualitative source, while several categories were represented across three or more forms of evidence. This pattern strengthens confidence that the final themes emerged from converging data rather than source-specific interpretations. The matrix also illustrates the complementary contribution of questionnaires, interviews, observations, platform records, and teacher perspectives in documenting and interpreting the qualitative manifestations of motivational withdrawal within exam-oriented vocational English-learning contexts.
